# Transcriptional and Metabolomic Analysis of L-Arginine/Nitric Oxide Pathway in Inflammatory Bowel Disease and Its Association with Local Inflammatory and Angiogenic Response: Preliminary Findings

**DOI:** 10.3390/ijms21051641

**Published:** 2020-02-28

**Authors:** Małgorzata Krzystek-Korpacka, Mariusz G. Fleszar, Iwona Bednarz-Misa, Łukasz Lewandowski, Izabela Szczuka, Radosław Kempiński, Katarzyna Neubauer

**Affiliations:** 1 Department of Medical Biochemistry, Wroclaw Medical University, 50-368 Wrocław, Poland; mariusz.fleszar@gmail.com (M.G.F.); iwona.bednarz-misa@umed.wroc.pl (I.B.-M.); lukasz.lewandowski@umed.wroc.pl (Ł.L.); izabela.szczuka@umed.wroc.pl (I.S.); 2 Department of Gastroenterology and Hepatology, Wroclaw Medical University, 50-556 Wrocław, Poland; radoslaw.kempinski@umed.wroc.pl (R.K.); katarzyna.neubauer@umed.wroc.pl (K.N.)

**Keywords:** Crohn’s disease, ulcerative colitis, arginase (ARG), nitric oxide synthase (NOS), arginine *N*-methyltransferase (PRMT), dimethylarginine dimethylaminohydrolase (DDAH), asymmetric dimethylarginine (ADMA), symmetric dimethylarginine (SDMA), L-citrulline, dimethylamine (DMA)

## Abstract

L-arginine/nitric oxide pathway in Crohn’s disease (CD) and ulcerative colitis (UC) is poorly investigated. The aim of current study is to quantify pathway serum metabolites in 52 CD (40 active), 48 UC (33 active), and 18 irritable bowel syndrome patients and 40 controls using mass spectrometry and at determining mRNA expression of pathway-associated enzymes in 91 bowel samples. Arginine and symmetric dimethylarginine decreased (*p* < 0.05) in active-CD (129 and 0.437 µM) compared to controls (157 and 0.494 µM) and active-UC (164 and 0.52 µM). Citrulline and dimethylamine increased (*p* < 0.05) in active-CD (68.7 and 70.9 µM) and active-UC (65.9 and 73.9 µM) compared to controls (42.7 and 50.4 µM). Compared to normal, CD-inflamed small bowel had downregulated (*p* < 0.05) arginase-2 by 2.4-fold and upregulated dimethylarginine dimethylaminohydrolase (*DDAH*)-2 (1.5-fold) and arginine N-methyltransferase (*PRMT*)-2 (1.6-fold). Quiescent-CD small bowel had upregulated (*p* < 0.05) arginase-2 (1.8-fold), *DDAH1* (2.9-fold), *DDAH2* (1.5-fold), *PRMT1* (1.5-fold), *PRMT2* (1.7-fold), and *PRMT5* (1.4-fold). Pathway enzymes were upregulated in CD-inflamed/quiescent and UC-inflamed colon as compared to normal. Compared to inflamed, quiescent CD-colon had upregulated *DDAH1* (5.7-fold) and ornithine decarboxylase (1.6-fold). Concluding, the pathway is deregulated in CD and UC, also in quiescent bowel, reflecting inflammation severity and angiogenic potential. Functional analysis of PRMTs and DDAHs as potential targets for therapy is warranted.

## 1. Introduction

**Figure 1:**
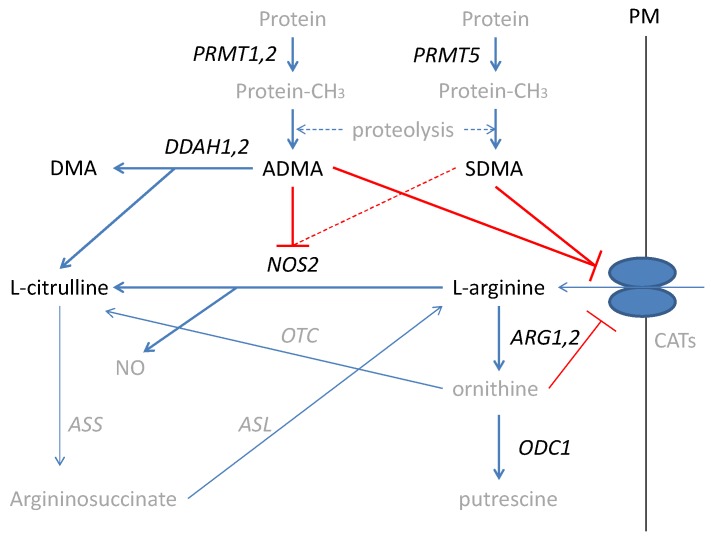
Simplified scheme of L-arginine/NO pathway. Pathway players analyzed in the current study are written in black: metabolites in straight font and enzymes in italics. Inhibition is marked in red (as a dashed line if the effect is weak). PRMT, arginine N-methyltransferase; PM, plasma membrane; CATs, cationic amino acid transporters; DDAH, dimethylarginine dimethylaminohydrolase; DMA, dimethylamine; ADMA, asymmetric dimethylarginine; SDMA, symmetric dimethylarginine; NOS, nitric oxide synthase; NO, nitric oxide; ARG, arginase; ODC, ornithine decarboxylase; ASS, argininosuccinate synthase; ASL, argininosuccinate lyase; OTC, ornithine transcarbamylase; protein-CH_3_, methylated proteins.

Inflammatory bowel diseases (IBD), including ulcerative colitis (UC) and Crohn’s disease (CD), are life-long, devastating, complex, and costly conditions of uncertain pathogenesis [1,2] and increasing incidence [3,4]. IBD-related chronic inflammation increases the probability of neoplastic transformation in the altered colon architecture leading to colorectal cancer (CRC) [5,6], with a prominent role attributed to nitric oxide (NO) [7,8,9]. The NO in the inflamed bowel is generated mostly by an inducible nitric oxide synthase (NOS2) from L-arginine (further referred to as arginine). Arginine is a non-essential amino acid synthesized in the liver as well as in the gut–kidney axis, which, under stress conditions, becomes conditionally essential [10]. Arginine is also used in the synthesis of urea and ornithine by arginases (ARG) with ornithine serving as a substrate for ornithine decarboxylase (ODC) in the polyamines synthesis pathway [11,12]. NO formation can be inhibited by arginine structural analogs—asymmetric and symmetric dimethylarginines (ADMA and SDMA, respectively). Methylarginines are derived from degraded proteins, previously methylated by a family of arginine *N*-methyltransferases (PRMTs), of which type I PRMTs (e.g., PRMT1 and PRMT2) lead to ADMA and type II PRMTs (e.g., PRMT5) yield SDMA. While SDMA is mostly excreted with urine, the ADMA pool is regulated by the activity of dimethylarginine dimethylaminohydrolases (DDAHs), which degrade ADMA, yielding citrulline and dimethylamine (DMA) [10] (Figure 1).

There is a growing interest in modulating the L-arginine/NO pathway as a chemopreventive strategy in CRC. Combination chemoprevention with ODC1 inhibitors has shown very promising results [13,14,15]. However, targeting ODC1 in IBD is controversial as polyamines are necessary for intestinal healing and gut health in general [16,17]. Modulating arginine availability has recently gained attention as well. Its deprivation is viewed as a way to starve the arginine auxotrophic tumors [11,18]. In IBD, on the contrary, arginine supplementation is advocated both for chemoprevention of colitis-associated carcinogenesis and for reduction of colonic inflammation [19,20].

In the light of the emerging relevance of the L-arginine/NO pathway for therapeutic interventions, a better understanding of its key players in IBD is needed. Concerning systemic concentrations of pathway metabolites, an elevation in ADMA and SDMA in IBD has been shown [21] but their association with local expression of the relevant pathway enzymes—DDAHs and PRMTs—remains unknown. Serum citrulline is reportedly decreased in IBD [22] or unaltered [23] while arginine has been found unaltered [21,24] but its local availability reduced and accompanied by decreased *ARG1* [24] and increased *ARG2* [24] and *NOS2* expression [24,25]. There is a paucity of data concerning the status of DDAHs and PRMTs, of which the latter have recently emerged as the regulators of global gene expression [26] as well as protein stability and function [27]. Previously, only PRMT5 upregulation has been linked with intestinal inflammation in animal models of the disease [28]. Therefore, the aim of this study is a comprehensive analysis of expression levels of key enzymes from the L-arginine/NO pathway in the context of the disease activity, local inflammatory and angiogenic response, and systemic concentrations of pathway metabolites in order to gain a better understanding of pathway status and to identify potential targets for intervention. For this purpose, the expression of *ARG1*, *ARG2*, *NOS2*, *ODC1*, *PRMT1*, *PRMT2*, *PRMT5*, *DDAH1*, and *DDAH2* in normal, quiescent, and inflamed bowel tissue has been quantified and referred to the disease severity, the expression of local indices of inflammation, angiogenesis, proliferation, cell cycle progression, and apoptosis and to the systemic concentrations of arginine, citrulline, ADMA, SDMA, and DMA.

## 2. Results

### 2.1. Systemic Concentrations of L-arginine/NO Pathway Metabolites

#### 2.1.1. L-arginine/NO Pathway Metabolites in IBD and in Irritable Bowel Syndrome (IBS)

The IBD patients had significantly higher citrulline and higher DMA concentrations than healthy controls. As compared to IBS patients, they had lower citrulline and higher SDMA concentrations. As compared to controls, IBS patients had higher citrulline and DMA and lower SDMA concentrations. There were no significant differences in ADMA between groups (Table 1). Individual distribution of metabolites in a study population is presented in the Supplementary Materials as Figure S1.

#### 2.1.2. L-Arginine/NO Pathway Metabolites and IBD Phenotype and Clinical Activity

Detailed analysis, including the disease phenotype and clinical activity, showed that patients with active CD had the lowest arginine concentrations, significantly so as compared to healthy controls and active UC. They also had higher citrulline concentrations than controls but lower than IBS patients, lower concentrations of SDMA than controls but higher than IBS patients, and higher DMA concentrations than controls and patients with inactive CD. Inactive CD was associated with elevated citrulline as compared to controls and SDMA as compared to IBS patients as well as with DMA concentrations lower than in active CD, UC, and IBS and higher than in controls. Patients with active UC had arginine higher than those with active CD had. They also had higher citrulline than controls but it was lower than in patients with IBS or inactive UC. Their SDMA concentration was higher than in patients with active CD or IBS and their DMA was higher than in patients with inactive CD or controls. Patients with inactive UC had significantly higher citrulline than controls and patients with active disease and higher DMA than controls (Table 2). Individual distribution of metabolites in study subgroups is presented in the Supplementary Materials as Figure S2.

Of the evaluated metabolites, the concentrations of arginine (*r* = −0.39, *p* = 0.006), SDMA (*r* = −0.32, *p* = 0.021), and citrulline (*r* = −0.27, *p* = 0.048) in CD patients inversely correlated with CDAI while those of ADMA (*p* = 0.523) and DMA (*p* = 0.153) showed no significant correlation. In UC, citrulline was the only metabolite correlated with RI (*r* = −0.38, *p* = 0.010); other metabolites showed no significant correlation (*p* = 0.132 for SDMA, *p* = 0.578 for DMA, *p* = 0.991 for ADMA, and *p* = 0.861 for arginine).

#### 2.1.3. L-arginine/NO Pathway Metabolites and the Endoscopic Activity of UC

Except for arginine, significantly different between patients with endoscopically inactive and the most active disease, systemic metabolite concentrations were not related to the endoscopic activity of UC expressed in terms of the Mayo endoscopic activity score (Table 3). Arginine positively correlated with endoscopic score (ρ = 42, *p* = 0.015).

#### 2.1.4. Interrelationship between L-arginine/NO Pathway Metabolites

**Figure 2:**
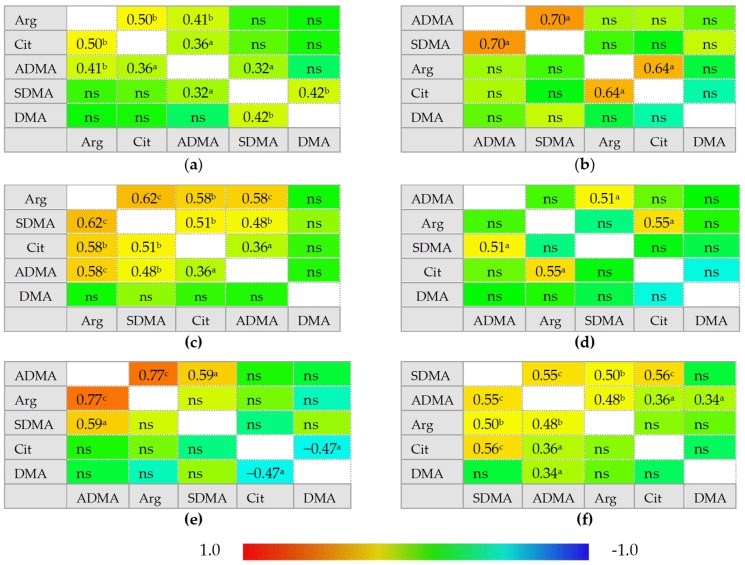
Correlogram for L-arginine/NO pathway metabolites: (**a**) in patients with active Crohn’s disease; (**b**) in patients with inactive Crohn’s disease; (**c)** in patients with active ulcerative colitis; (**d**) in patients with inactive ulcerative colitis; (**e**) in patients with irritable bowel syndrome; (**f**) in healthy controls. Results presented as correlation coefficients *r* and analyzed on log-transformed data using Pearson’s correlation test; ^a^ *p* < 0.05; ^b^ *p* < 0.01; ^c^ *p* < 0.001; ^d^ *p* < 0.0001; ns, non-significant; Arg, arginine; Cit, citrulline; ADMA, asymmetric dimethylarginine; SDMA, symmetric dimethylarginine; DMA, dimethylarginine.

In healthy individuals, ADMA and SDMA are interrelated with each other and with arginine and citrulline concentrations but there is no correlation between citrulline and arginine. In IBS, neither ADMA nor SDMA correlated with citrulline, which instead becomes inversely related to DMA concentrations. In inactive CD and UC, ADMA is correlated exclusively with SDMA and arginine becomes positively correlated with citrulline, while a tendency towards a negative association between DMA and citrulline could be found, especially in inactive UC. Active CD can be distinguished from inactive disease by weaker ADMA and SDMA correlation, positive association between SDMA and DMA, and by ADMA correlation with both citrulline and arginine. Active UC, in turn, can be distinguished from inactive disease by arginine and citrulline correlation with both ADMA and SDMA. Active CD and UC differ as well as there is no SDMA correlation with arginine, citrulline, or SDMA in active UC (Figure 2).

In multiple regression, citrulline was an independent predictor of serum arginine concentrations in active (r_p_ = 0.50, *p* = 0.001) and inactive CD (r_p_ = 0.64, *p* = 0.045), explaining, respectively, 25% (R^2^ = 0.253; F = 12.5, *p* = 0.001) and 41% (R^2^ = 0.414; F = 5.7, *p* = 0.045) in its variability. Citrulline was also an independent predictor of arginine in inactive UC (r_p_ = 0.55, *p* = 0.040), explaining 31% in amino acid variability (R^2^ = 0.306; F = 5.3, *p* = 0.040), but in active disease, ADMA (r_p_ = 0.51, *p* = 0.007) and SDMA (r_p_ = 0.53, *p* = 0.005) were independently associated with arginine concentrations, explaining together 56% in its variability (R^2^ = 0.561; F = 16, *p* < 0.0001).

ADMA (r_p_ = 0.77, *p* < 0.001) was an independent predictor of serum arginine concentrations in IBS patients, explaining 60% in its variability (R^2^ = 0.596; F = 23.6, *p* < 0.001). In healthy controls, arginine concentrations were predicted by SDMA (r_p_ = 0.50, *p* = 0.001), the variability in which was responsible for 25% of arginine variability (R^2^ = 0.246; F = 12.4, *p* = 0.001).

### 2.2. L-arginine/NO Pathway-Associated Enzymes in IBD—Transcriptional Analysis

#### 2.2.1. L-arginine/NO Pathway-Associated Enzymes in Inflamed and Quiescent Small Bowel

Quiescent tissue derived from CD patients occurred to have significantly higher *DDAH1* (by 2.9-fold) and *DDAH2* (by 1.5-fold) as well as *PRMT1* (by 1.5-fold), *PRMT2* (by 1.7-fold)*,* and *PRMT5* (by 1.4-fold) expression as compared to normal small bowel tissue from UC patients. Inflamed tissue had lower expression of *ARG2* (by 4.2-fold) than quiescent tissue of CD patients and lower *ARG2* (by 2.4-fold) but higher *DDAH2* (by 1.5-fold) and *PRMT2* (by 1.6-fold) than normal small bowel tissue from UC patients (Table 4). Individual distribution of enzyme transcripts in inflamed and quiescent as compared to normal small bowel is presented in the Supplementary Materials as Figure S3.

Gene expression level in inflamed tissue correlated with clinical disease activity index CDAI in case of *PRMT1* (ρ = 0.78, *p* = 0.001, *n* = 14), *PRMT2* (ρ = 0.54, *p* = 0.047, *n* = 14), and *PRMT5* (ρ = 0.71, *p* = 0.005, *n* = 14) and displayed such tendency in case of *ODC1* expression (ρ = 0.46, *p* = 0.095, *n* = 14).

Gene expression level in quiescent tissue from CD patients tended to positively correlate with CDAI in case of *PRMT2* (ρ = 0.47, *p* = 0.088, *n* = 14) but was significantly correlated in case of *ARG1* (ρ = 0.62, *p* = 0.019, *n* = 14) and *DDAH2* (ρ = 0.68, *p* = 0.008, *n* = 14).

**Figure 3:**
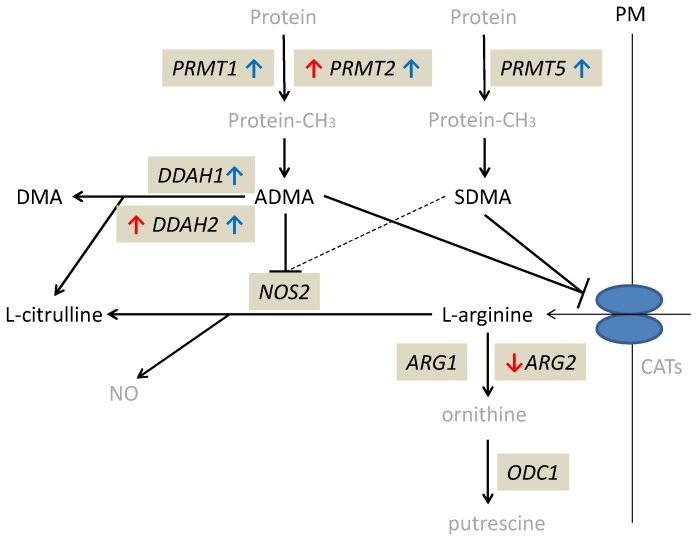
Alterations in expression of L-arginine/NO pathway enzymes in inflamed and quiescent tissue from patients with Crohn’s disease as compared to normal small bowel tissue derived from patients with ulcerative colitis. Changes observed in inflamed small bowel are marked by red arrows and changes observed in quiescent small bowel by blue arrows. Pathway players analyzed in the current study are written in black: metabolites in straight font and enzymes in italics. Inhibition is marked as T-arrows (as a dashed line if the effect is weak). PRMT, arginine N-methyltransferase; PM, plasma membrane; CATs, cationic amino acid transporters; DDAH, dimethylarginine dimethylaminohydrolase; DMA, dimethylamine; ADMA, asymmetric dimethylarginine; SDMA, symmetric dimethylarginine; NOS, nitric oxide synthase; NO, nitric oxide; ARG, arginase; ODC, ornithine decarboxylase; protein-CH_3_, methylated proteins.

The alterations in expression patterns of L-arginine/NO pathway enzymes in inflamed and quiescent tissue from CD patients as compared to normal small bowel are summarized in Figure 3.

#### 2.2.2. L-arginine/NO Pathway-Associated Enzymes in Inflamed and Quiescent Large Bowel

Except for *DDAH1*, the expression of which was significantly higher only in quiescent tissue, all evaluated genes were significantly upregulated in quiescent and inflamed bowel tissue, both in CD and UC. Quiescent tissue had higher expression of *DDAH1* than inflamed tissue from CD (by 5.7-fold) and UC patients (by 3.8-fold), higher *DDAH2* (by 1.7-fold) and *PRMT2* (by 1.2-fold) expression than inflamed tissue from UC patients and higher *ODC1* than inflamed tissue from CD patients (by 1.6-fold). In turn, *NOS2* expression in quiescent tissue was lower than that in inflamed tissue from UC (by 10.8-fold) but not CD patients. Inflamed tissue from CD patients had significantly upregulated expression of *DDAH2* (by 1.7-fold) and downregulated expression of *NOS2* (by 37.9-fold) as compared to inflamed tissue from UC patients (Table 5). Individual distribution of enzyme transcripts in inflamed and quiescent as compared to normal large bowel is presented in the Supplementary Materials as Figure S4.

**Figure 4:**
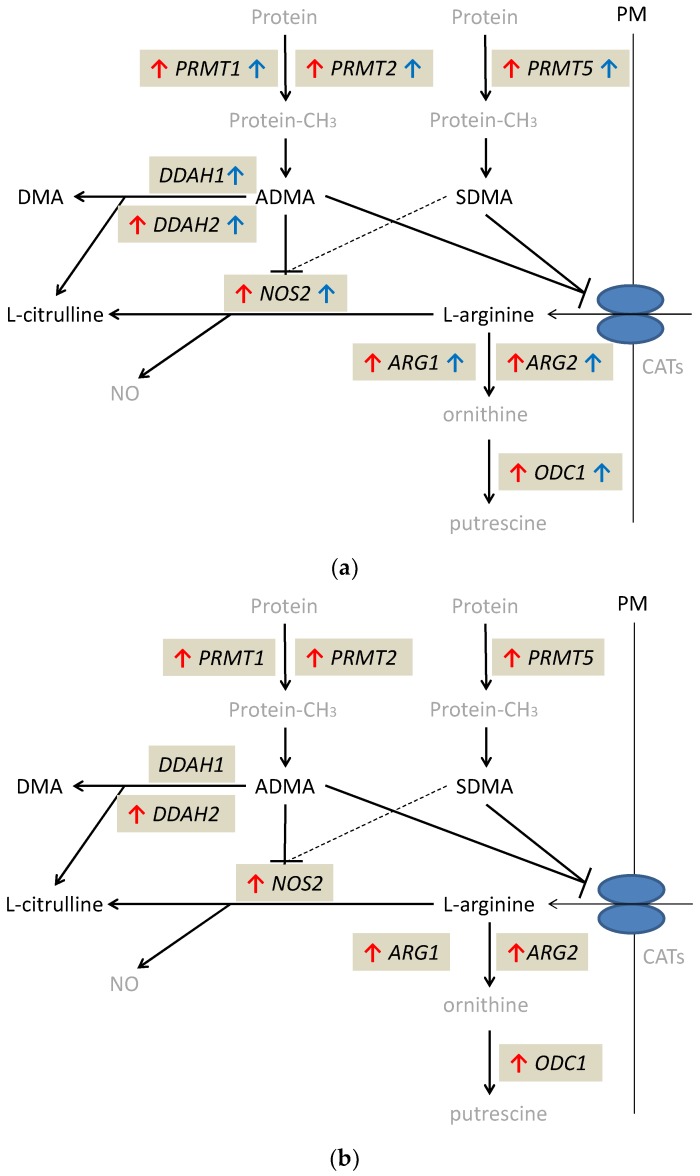
Alterations in expression of L-arginine/NO pathway enzymes: **(a)** in inflamed and quiescent tissue from patients with Crohn’s disease as compared to normal large bowel tissue derived from patients undergoing polypectomy; **(b)** in inflamed tissue from patients with ulcerative colitis as compared to normal large bowel tissue derived from patients undergoing polypectomy. Changes observed in inflamed large bowel are marked by red arrows and changes observed in quiescent large bowel by blue arrows. Pathway players analyzed in the current study are written in black: metabolites in straight font and enzymes in italics. Inhibition is marked as T-arrows (as a dashed line if the effect is weak). PRMT, arginine N-methyltransferase; PM, plasma membrane; CATs, cationic amino acid transporters; DDAH, dimethylarginine dimethylaminohydrolase; DMA, dimethylamine; ADMA, asymmetric dimethylarginine; SDMA, symmetric dimethylarginine; NOS, nitric oxide synthase; NO, nitric oxide; ARG, arginase; ODC, ornithine decarboxylase; protein-CH_3_, methylated proteins.

The alterations in expression patterns of L-arginine/NO pathway enzymes in inflamed and quiescent tissue from IBD patients as compared to normal large bowel are summarized in Figure 4. 

In CD patients, gene expression level in inflamed tissue correlated with clinical disease activity index CDAI in case of *PRMT2* (ρ = −0.73, *p* = 0.026, *n* = 9) and displayed such tendency in case of *DDAH1* expression (ρ = −0.63, *p* = 0.070, *n* = 9). For all genes, the association tended to be negative except for *ARG1* displaying positive association (ρ = 0.61, *p* = 0.081, *n* = 9) and *NOS2* showing no association.

Gene expression level in inflamed tissue from UC patients correlated with clinical disease activity index RI in case of *ARG1* (ρ = 0.66, *p* = 0.019, *n* = 12), *ARG2* (ρ = 0.86, *p* < 0.001, *n* = 12), *DDAH1* (ρ = 0.75, *p* = 0.005, *n* = 12), *PRMT1* (ρ = 0.59, *p* = 0.043, *n* = 12), and *PRMT2* (ρ = 0.64, *p* = 0.024, *n* = 12). *PRMT5* expression and displayed a similar tendency (ρ = 0.51, *p* = 0.094, *n* = 12).

#### 2.2.3. Interrelationship between Expression Levels of L-arginine/NO Pathway-Associated Enzymes in the Small and Large Bowel

**Figure 5:**
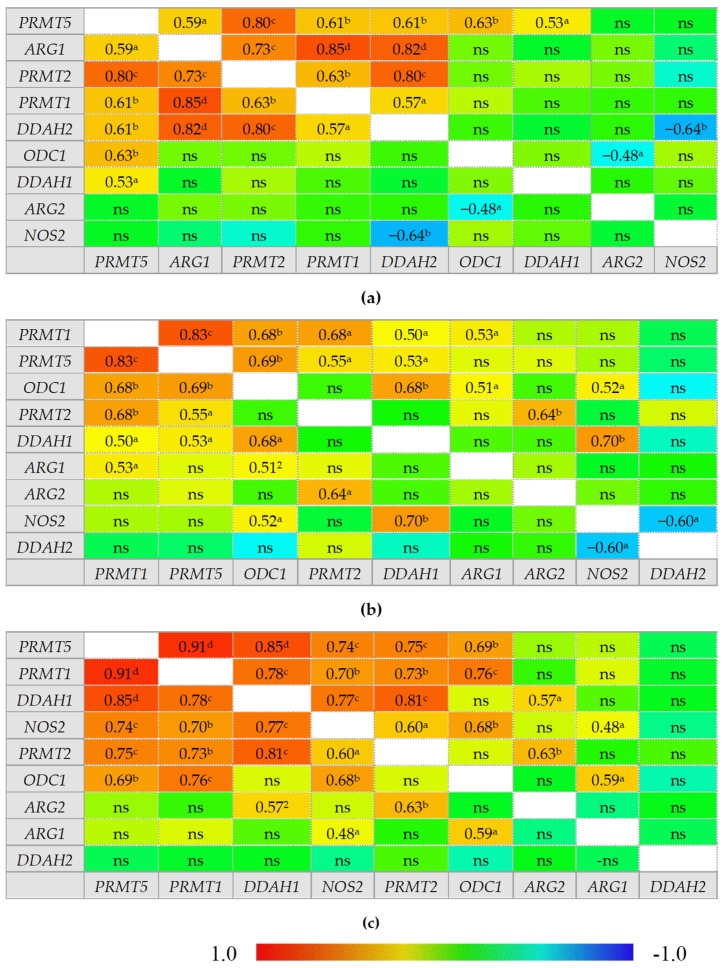
Correlogram for genes encoding L-arginine/NO pathway enzymes: (**a**) in quiescent small bowel from patients with Crohn’s disease; (**b**) in inflamed small bowel from patients with Crohn’s disease; (**c**) in normal small bowel from patients with ulcerative colitis. Results presented as correlation coefficients *r* and analyzed on log-transformed data using Pearson’s correlation test; ^a^ *p* < 0.05; ^b^ *p* < 0.01; ^c^ *p* < 0.001; ^d^ *p* < 0.0001; ns, non-significant; ARG, arginase; DDAH, dimethylarginine dimethylaminohydrolase, NOS, nitric oxide synthase; ODC, ornithine decarboxylase; PRMT, arginine N-methyltransferase.

**Figure 6:**
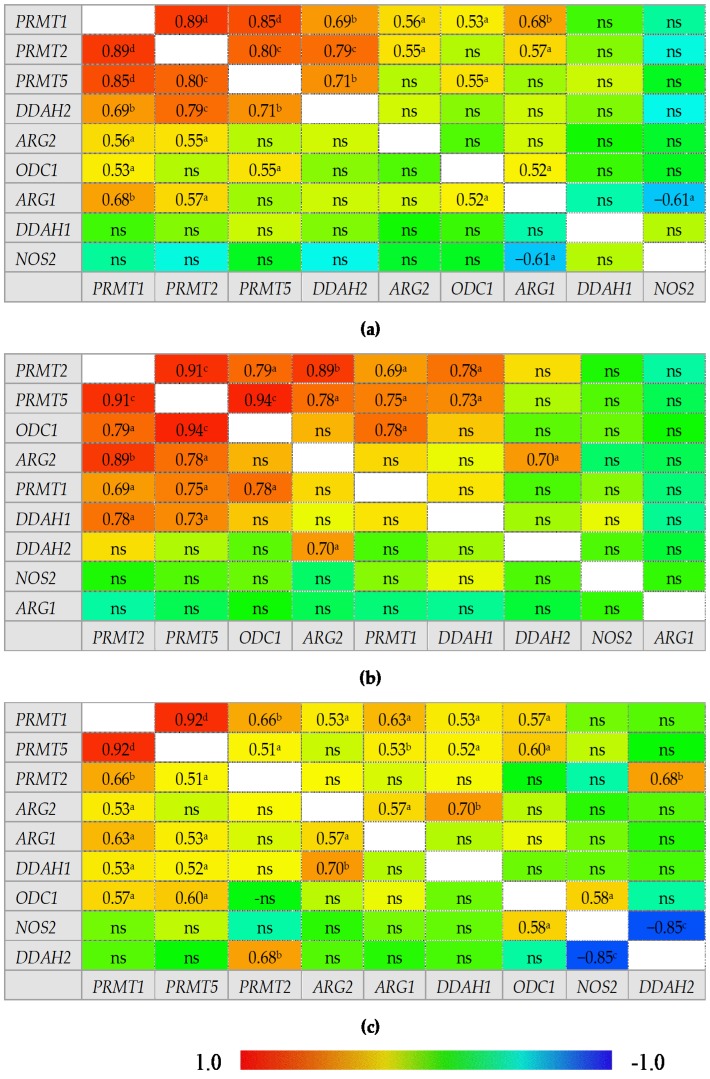
Correlogram for genes encoding L-arginine/NO pathway enzymes: (**a**) in quiescent large bowel from patients with Crohn’s disease; (**b**) in inflamed large bowel from patients with Crohn’s disease; (**c**) in inflamed large bowel from patients with ulcerative colitis. Results presented as correlation coefficients *r* and analyzed on log-transformed data using Pearson’s correlation test; ^a^ *p* < 0.05; ^b^ *p* < 0.01; ^c^ *p* < 0.001; ^d^ *p* < 0.0001; ns, non-significant; ARG, arginase; DDAH, dimethylarginine dimethylaminohydrolase, NOS, nitric oxide synthase; ODC, ornithine decarboxylase; PRMT, arginine N-methyltransferase.

An overview of correlation patterns in the small (Figure 5) and large bowel (Figure 6) shows a strong association between expression level of all three *PRMTs* and rather lack of interrelationship between *DDAH1* and *DDAH2* and between *ARG1* and *ARG2*.

In normal small bowel, *DDAH1* expression was tightly associated with that of *PRMTs* but the association was weakened or lost in inflamed and quiescent tissue. Moreover, *PRMTs* in quiescent tissue became associated with *DDAH2* expression. In normal small bowel, *NOS2* expression was positively correlated with that of *PRMTs*, *ODC1*, and *DDAH1* while in the inflamed tissue only the association with *ODC1* and *DDAH1* remained and in quiescent tissue *NOS2* was correlated with neither. Instead, an inverse relationship with *DDAH2* could be observed in inflamed and quiescent small bowel. Both ARGs were poorly related with the expression of remaining enzymes in normal and inflamed tissue with moderate positive correlation with *ODC1* and *PRMT1* or *NOS2* for *ARG1* and with *PRMT2* and *DDAH1* for *ARG2*. However, in quiescent small bowel, *ARG1* expression became closely related to that of *PRMTs* and *DDAH2* while that of *ARG2* became inversely related with *ODC1*.

In the large bowel, CD-associated inflammation was distinguished from UC by a tight association between expression of *ODC1* and *PRMTs*, by *ARG2* association with *DDAH2* instead of *DDAH1*, and by lack of correlations for *ARG1*, which, in UC inflammation, was related to the expression of *PRMTs*. In addition, *NOS2* displayed a positive correlation with *ODC1* and a strong inverse correlation with *DDAH2* exclusively in UC-associated inflammation. In turn, inflamed from quiescent bowel from CD patients was distinguished by stronger positive correlation between *ODC1* and *PRMTs*, positive correlation between *PRMTs* and *DDAH1* instead of *DDAH2*, and by lack of positive correlation between *ARG1* and *PRMTs* or *ODC1* and negative between *ARG1* and *NOS2*.

#### 2.2.4. Expression of L-arginine/NO Pathway-Associated Enzymes in the Bowel and Local Inflammatory and Angiogenic Response, Hypoxia, Proliferation, and Apoptosis

The correlation between expression of L-arginine/NO pathway-associated enzymes and the bowel expression of mediators of inflammation (*IL1B*, *TNFA*, *CCL2*, *CCL3*, *CCL4*) and angiogenesis (*VEGFA*, *IL8*, *FGF2*) and the markers of proliferation (*PCNA*), hypoxia (*HIF1A*), and cell cycle and apoptosis (*BAX*, *CDKN1A*, *TP53*) was examined. For the purpose of correlation analysis, two groups, distinguished based on the presence of inflammation and not IBD location or phenotype, were formed. An “inflamed” group included samples of inflamed large and small bowel from CD patients and inflamed large bowel from UC patients, whereas a “non-inflamed” group included samples of quiescent small and large bowel from CD patients and normal small bowel from UC patients.

**Figure 7:**
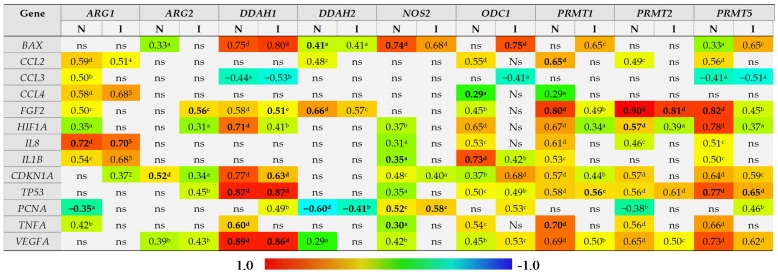
Correlogram of associations between the expression level of genes encoding L-arginine/NO pathway enzymes and genes encoding mediators of inflammation and angiogenesis and markers of proliferation, hypoxia, and cell cycle progression and apoptosis in inflamed and non-inflamed bowel tissue. An “inflamed” group includes samples of inflamed large and small bowel from patients with Crohn’s disease and inflamed large bowel from patients with ulcerative colitis, whereas a “non-inflamed” group includes samples of quiescent small and large bowel from patients with Crohn’s disease and normal small bowel from patients with ulcerative colitis. Results presented as correlation coefficients *r* and analyzed on log-transformed data using Pearson’s correlation test separately for inflamed (I; *n* = 40) and non-inflamed (*N*; *n* = 50) bowel tissue; ^a^ *p* < 0.05; ^b^ *p* < 0.01; ^c^ *p* < 0.001; ^d^ *p* < 0.0001; ns, non-significant. Variables retained as independent predictors (a stepwise method of least squares multiple regression analysis) in non-inflamed or inflamed bowel tissue are written in bold. ARG, arginase; DDAH, dimethylarginine dimethylaminohydrolase, NOS, nitric oxide synthase; ODC, ornithine decarboxylase; PRMT, arginine N-methyltransferase; BAX, Bcl-2-associated X protein; CCL2–4, chemokine (C-C motif) ligand 2–4; FGF2, basic fibroblast growth factor 2; HIF1A, hypoxia-inducible factor 1α, IL, interleukin; CDKN1A, cyclin-dependent kinase inhibitor 1; TP53, tumor protein p53; PCNA, proliferating cell nuclear antigen; TNFA, tumor necrosis factor α; VEGFA, vascular endothelial growth factor A.

The level of *ARG1* expression in the bowel was positively correlated with expression of inflammatory mediators *IL1B*, *TNFA*, *CCL2–4*, although with *TNFA* and *CCL3* exclusively in non-inflamed tissue, with markers of angiogenesis *HIF1A*, *IL8*, and *FGF2* but, except for *IL8*, exclusively in non-inflamed tissue, and with *CDKN1A* expression in inflamed bowel tissue. A negative correlation was observed for *PCNA* in non-inflamed tissue (Figure 7). Of these, the expression of *IL8* (r_p_ = 0.72, *p* < 0.0001) and *PCNA* (r_p_ = −0.34, *p* = 0.019) was independently from other variables associated with *ARG1* expression in non-inflamed bowel and explained 58% (R^2^ = 0.578; F = 32.1, *p* < 0.0001) in *ARG1* variability. In the inflamed bowel, *IL8* was the sole independent predictor of *ARG1* expression (r_p_ = 0.70, *p* < 0.0001) explaining 49% (R^2^ = 0.487; F = 36.1, *p* < 0.0001) in *ARG1* variability.

*ARG2* expression was positively correlated with *CDKN1A* as well, both in inflamed and non-inflamed tissue, and with *TP53* in inflamed tissue. In turn, its correlation with markers of angiogenesis, *VEGFA*, *FGF2*, and *HIF1A* could be observed rather in inflamed than non-inflamed tissue. In turn, in non-inflamed tissue *ARG2* was weakly positively correlated with *BAX* expression (Figure 7). Of these, the expression of *FGF2* (r_p_ = 0.56, *p* < 0.001) in inflamed and *CDKN1A* (r_p_ = 0.52, *p* < 0.001) in non-inflamed bowel tissue were independently from other variables associated with *ARG2* expression, explaining, respectively, 31% (R^2^ = 0.314; F = 17.4, *p* < 0.001) and 28% (R^2^ = 0.275; F = 18.2, *p* < 0.001) in its variability.

*DDAH1* expression positively correlated with markers of angiogenesis *VEGFA*, *FGF2*, and *HIF1A*, and with *TP53*, *CDKN1A*, and *BAX* and negatively with *CCL3*, both in inflamed and non-inflamed bowel tissue, and with *PCNA* in inflamed and *TNFA* in non-inflamed tissue (Figure 7). Of these, *HIF1A* (r_p_ = −0.51, *p* < 0.001), *TP53* (r_p_ = 0.74, *p* < 0.0001), *TNFA* (r_p_ = −0.33, *p* = 0.023), and *VEGFA* (r_p_ = 0.71, *p* < 0.0001) were independently associated with *DDAH1* expression in non-inflamed tissue, explaining 91% in its variability (R^2^ = 0.905; F = 107.6, *p* < 0.0001). In inflamed bowel, *FGF2* (r_p_ = −0.38, *p* = 0.021), *CDKN1A* (r_p_ = −0.46, *p* = 0.005), *TP53* (r_p_ = 0.55, *p* < 0.001), and *VEGFA* (r_p_ = 0.51, *p* = 0.001) were independently associated with *DDAH1* expression, explaining 83% in its variability (R^2^ = 0.832; F = 43.4, *p* < 0.0001).

*DDAH2* expression positively correlated with *BAX* and *FGF2* and inversely with *PCNA* in both inflamed and non-inflamed tissue and additionally with *CCL2* and *VEGFA* in non-inflamed bowel (Figure 7). Of those, *FGF2* (r_p_ = 0.62, *p* < 0.001), *BAX* (r_p_ = −0.39, *p* = 0.019), and *PCNA* (r_p_ = −0.44, *p* = 0.007) were independently associated with *DDAH2* expression in non-inflamed bowel, explaining 74% in gene variability (R^2^ = 0.743; F = 32.8, *p* < 0.0001). In inflamed bowel, *PCNA* (r_p_ = −0.55, *p* = 0.002) was a sole predictor of *DDAH2* expression level, explaining 30% of its variability (R^2^ = 0.303; F = 11.3, *p* = 0.002).

*NOS2* expression positively correlated with *BAX*, *HIF1A*, *IL8*, *IL1B*, *CDKN1A*, *TP53*, *PCNA*, *TNFA*, and *VEGFA* in non-inflamed and with *BAX*, *CDKN1A*, and *PCNA* in inflamed bowel (Figure 7). Of those, *BAX* (r_p_ = 0.66, *p* < 0.0001), *IL1B* (r_p_ = 0.58, *p* < 0.001), *PCNA* (r_p_ = 0.49, *p* = 0.003), and *TNFA* (r_p_ = −0.35, *p* = 0.039) were independent predictors of *NOS2* expression, explaining 74% in its variability in non-inflamed tissue (R^2^ = 0.744; F = 23.9; *p* < 0.0001). In inflamed bowel, *PCNA* (r_p_ = 0.79, *p* < 0.0001) was a sole predictor of *NOS2* expression level, explaining 62% of its variability (R^2^ = 0.620; F = 42.4, *p* < 0.0001).

*ODC1* expression positively correlated with all genes except for *BAX*, *CCL3*, and *PCNA* in non-inflamed tissue and positively with *BAX*, *IL1B*, *CDKN1A*, *TP53*, *PCNA*, and *VEGFA* and negatively with *CCL3* in inflamed tissue (Figure 7). Of those, *CCL4* (r_p_ = −0.35, *p* = 0.014) and *IL1B* (r_p_ = 0.74, *p* < 0.0001) were independent predictors of *ODC1* expression in non-inflamed bowel, explaining 59% in gene variability (R^2^ = 0.587; F = 33.3, *p* < 0.0001). In inflamed tissue, *BAX* (r_p_ = 0.75, *p* < 0.0001) was a sole predictor of *ODC1* expression level, explaining 57% of its variability (R^2^ = 0.566; F = 33.9, *p* < 0.0001).

*PRMT1* expression was positively correlated with all genes except for *BAX*, *CCL3*, and *PCNA* in non-inflamed tissue and positively with *BAX*, *FGF2*, *HIF1A*, *CDKN1A*, *TP53*, and *VEGFA* in inflamed tissue (Figure 7). Of those, *CCL2* (r_p_ = 0.30, *p* = 0.042), *FGF2* (r_p_ = 0.48, *p* < 0.001), and *TNFA* (r_p_ = 0.33, *p* = 0.021) were independent predictors of *PRMT1* expression in non-inflamed bowel, explaining 70% in gene variability (R^2^ = 0.702; F = 36.1, *p* < 0.0001). In inflamed tissue, *TP53* (r_p_ = 0.56, *p* < 0.001) was a sole predictor of *PRMT1* expression level, explaining 31% of its variability (R^2^ = 0.311; F = 17.1, *p* < 0.001).

*PRMT2* expression was positively correlated with *CCL2*, *FGF2*, *HIF1A*, *IL8*, *CDKN1A*, *TP53*, *TNFA*, and *VEGFA* and negatively with *PCNA* in non-inflamed tissue and with *FGF2*, *HIF1A*, *TP53*, and *VEGFA* in inflamed tissue (Figure 7). Of those, *FGF2* (r_p_ = 0.88, *p* < 0.0001) and *HIF1A* (r_p_ = −0.38, *p* = 0.008) were independently associated with *PRMT2* expression, explaining 84% in its variability in non-inflamed bowel (R^2^ = 0.843; F = 126.3, *p* < 0.0001). In inflamed tissue, *FGF2* (r_p_ = 0.81, *p* < 0.0001) was a sole predictor of *PRMT2* expression level, explaining 65% of its variability (R^2^ = 0.650; F = 70.4, *p* < 0.0001).

*PRMT5* expression was positively correlated with all genes except for *CCL4* and *PCNA* and negatively with *CCL3* in non-inflamed tissue and positively with *BAX*, *FGF2*, *HIF1A*, *CDKN1A*, *TP53*, *PCNA*, and *VEGFA* and negatively with *CCL3* in inflamed bowel (Figure 7). Of those, *FGF2* (r_p_ = 0.61, *p* < 0.0001) and *TP53* (r_p_ = 0.47, *p* < 0.001) were independently associated with *PRMT5* expression, explaining 74% in its variability in non-inflamed bowel (R^2^ = 0.744; F = 68.4, *p* < 0.0001). In inflamed tissue, *TP53* (r_p_ = 0.65, *p* < 0.0001) was a sole predictor of *PRMT5* expression level, explaining 43% of its variability (R^2^ = 0.428; F = 28.4, *p* < 0.0001).

### 2.3. Expression of L-arginine/NO Pathway-Associated Enzymes in the Small and Large Bowel and Systemic Concentrations of Pathway Metabolites

Paired blood samples for metabolomic analysis and tissue samples (1–3 per patient, together 60 samples) for transcriptomic analysis were available for 28 patients, in whom correlation analysis was conducted.

The DDAH enzymes degrade ADMA into citrulline and DMA. While there was no correlation between their expression and systemic ADMA concentration, both *DDAHs* positively correlated with systemic concentrations of citrulline and DMA. *DDAH1* association was equally strong in the small and large bowel (ρ = 0.45, *p* = 0.009, *n* = 33 and ρ = 0.45, *p* = 0.019, *n* = 27) but it was significant only in non-inflamed tissue (ρ = 0.54, *p* < 0.001, *n* = 35). Correlation between *DDAH2* expression and citrulline was observed exclusively for the large bowel samples (ρ = 0.39, *p* = 0.044, *n* = 27), especially those derived from quiescent tissue (ρ = 0.71, *p* = 0.015, *n* = 11). In addition, *DDAH1* positively correlated with DMA, also in a non-inflamed tissue (ρ = 0.43, *p* = 0.010, *n* = 35), and *DDAH2*—in the large bowel (ρ = 0.44, *p* = 0.023, *n* = 27), especially in quiescent tissue (ρ = 0.86, *p* < 0.001, *n* = 11). Citrulline did not correlate with *NOS2* or with *ODC1* and *ARG2*, controlling alternative pathways for amino acid synthesis.

There was no significant correlation between *PRMT1* and *PRMT2* expression and systemic ADMA concentrations, except for a tendency towards inverse relationship for *PRMT1* in the small bowel (ρ = −0.33, *p* = 0.062, *n* = 33) and towards positive correlation for *PRMT2* in inflamed large bowel from UC patients (ρ = 0.58, *p* = 0.099, *n* = 9). No correlation between *PRMT5* expression and SDMA could be observed as well.

From among arginine-utilizing enzymes, *ARG1* expression tended to be inversely related to systemic arginine concentrations in CD-associated inflammation, more pronouncedly in the large bowel (ρ = 0.74, *p* = 0.052, *n* = 7). However, the expression of *ARG2* displayed a positive correlation with systemic arginine in UC patients (ρ = 0.59, *p* = 0.008, *n* = 19), both in samples derived from inflamed large colon (ρ = 0.67, *p* = 0.049, *n* = 9) and from normal small bowel (ρ = 0.65, *p* = 0.032, *n* = 11). Positive correlation was also observed between systemic arginine and *NOS2* expression in normal small bowel derived from UC patients (ρ = 0.64, *p* = 0.035, *n* = 11). No correlation between *ODC1* expression and arginine was found.

## 3. Discussion

In the light of limited data on PRMTs in IBD and the growing interest in their targeting as a chemoprevention and antineoplastic strategy, one of the most important findings of the current study is the observation of consistent upregulation of *PRMTs* in the small and large bowel of IBD patients. Previously, only Zheng et al. [28] investigated *PRMTs* expression in the bowel of DSS-treated mice, showing a high abundance of *PRMT1* and *PRMT5* and relatively low *PRMT2* and other isoenzymes as well as the upregulation of *PRMT5*, but not *PRMT1*, in intestinal inflammation. Interestingly, results presented here show that in human IBD, the *PRMTs* seem to be overexpressed, particularly in quiescent tissue, and in the small bowel exclusively so. *PRMT2* was an exception as it was also upregulated in the inflamed small bowel, whereas in the large bowel, it was more markedly overexpressed in quiescent tissue than inflamed derived from UC patients. Concerning the L-arginine/NO pathway, PRMTs act as negative effectors of NO synthesis by increasing the pool of ADMA and SDMA, inhibitors of NOSs, and competitors for arginine transporters [10]. Taking into account accelerated NO synthesis in IBD and its contribution to the disease pathogenesis [7], the up-regulation of PRMTs might be viewed as beneficial. However, the significance of PRMTs reaches far beyond the regulation of arginine availability and NO generation. The PRMT-mediated post-translational methylation of histones and non-histone proteins is a regulatory mechanism of protein expression, additionally implicated in modulating protein stability and function [26,27]. As evidenced here by *PRMTs*’ upregulation being accompanied by rather decreased systemic concentrations of dimethylarginines as well as by lack of direct correlation between the enzymes and their products, PRMTs might be rather loosely related with the L-arginine/NO pathway. On the other hand, however, their expression positively and moderately-to-strongly correlated with that of other pathway enzymes, e.g., *DDAHs*, and their concomitant up-regulation might contribute to the lack of ADMA accumulation. Nonetheless, PRMTs have been linked with regulation of immune and inflammatory responses, with PRMT5 being implicated in increasing NF-κB activity and the expression of downstream cytokines, chemokines, and growth factors [27] as well as in promoting HOXA9-dependent expression of leukocyte adhesion molecules on endothelial cells in response to TNF*α* stimulation [29]. Still, as a transcription cofactor, PRMT5 has been shown to repress *IL8* expression by mechanisms involving disabling SPT5-mediated transcriptional elongation [30]. PRMT1, in turn, has been implicated in the repression of inflammation by several mechanisms, namely, by preventing RelA from binding to target genes and methylation of TRAF6 [31], repressing IFN*γ*-induced MHC II transcription in macrophages [32], or by methylating PIAS1 and interfering with both IFN*γ* and *α* signaling [33]. Others, however, have demonstrated that PRMT1-mediated methylation of STAT1 is required for IFN*α*/*β*-induced transcription [34]. In line with the proinflammatory character of *PRMTs* overexpression, all *PRMTs* evaluated in the current study were correlated with local expression of inflammatory cytokines, more pronouncedly in quiescent tissue, and were positively correlated with the clinical activity disease indices, CDAI and RI. Correspondingly, Zheng et al. [28] demonstrated that in animal models of colitis, PRMT5 targeting reduces bowel expression of *IL1B*, *TNFA*, and *IL6*. Moreover, they showed PRMT5 inhibition to reduce IFNγ production by effector T-cells as well as to increase the number and improve functionality of Tregs, both in mice and humans, clearly linking PRMT5 with UC pathogenesis. As such, the authors have pointed at PRMT5 as a potential new target for pharmacotherapy in managing UC. It is therefore of particular interest that, in addition to inflammation, the results presented here link the expression of *PRMTs* with angiogenesis in IBD. Both in quiescent and inflamed tissue, the enzymes positively correlated with the expression of *HIF1A*, *IL8*, *VEGFA*, and *FGF2*, corroborating the recently discovered requirement for PRMT5 in HOXC10-mediated upregulation of *VEGFA* expression in endothelial cells [35]. Others have shown PRMT5 to be implicated in FGF2 methylation [36]. Therefore, the upregulation of *PRMTs* in quiescent bowel might play a positive role during healing processes following the disease flare. It might be initiated in order to restore homeostasis and repair intestinal architecture. However, the particularly close relationship with *FGF2* and co-expression during active inflammation may also implicate PRMTs’ contribution to aberrant angiogenesis and impaired healing, resulting in tissue fibrosis. A growing body of evidence indicates that epithelial-mesenchymal transition (EMT) is implicated in the pathogenesis of fibrosis in IBD. Accordingly, the presence of EMT markers has been demonstrated in fibrotic areas of IBD patients (reviewed in [37]). Recently, PRMT5 has been implicated in EMT by activating the Wnt/*β*-catenin pathway and inducing cancer cell migration [38]. PRMT5 might also contribute to hypermethylation of EMT-associated genes, the phenomenon observed in inflamed colonic mucosa in UC and speculated to contribute to the progression from ulcerative lesions to cancer [37]. In fact, PRMTs have been implicated in cancer initiation as well as progression, supporting cell proliferation, preventing apoptosis, and inducing angiogenesis and migration [26,39]. As such, their targeting in IBD patients may help to prevent neoplastic transformation. In particular, an oncogenic role associated with regulation of cell cycle proteins [40,41,42] as well as with facilitating splicing of genes retaining introns [43] has been attributed to PRMT5. Unlike other PRMTs, PRMT2 has been rather shown to prevent cancer cell proliferation [44]. Correspondingly, it was the only *PRMT* here negatively associated with the expression of proliferation marker PCNA. All investigated *PRMTs* were closely associated with *TP53* and *CDKN1A* expression, corroborating the notion of PRMT5 being a major pro-survival factor acting by regulating p53 and its downstream targets, including p21, encoded by *CDKN1A* [45].

As mentioned before, the upregulation of *PRMTs* in the studied patients was accompanied by concomitant overexpression of *DDAHs*. *DDAH1* was overexpressed exclusively in quiescent tissue, in both small and large bowel, while *DDAH2* also in inflamed ones. *DDAH2* expression in quiescent small bowel increased along with the patient’s CDAI and *DDAH1* expression in inflamed colon, along with RI. Intriguingly, although both isoenzymes catalyze, in theory, the same reaction, there was no correlation between their expression levels. Moreover, they displayed an inverse correlation pattern with *PRMTs*: in normal and inflamed small bowel, *PRMTs* correlated with *DDAH1*, whilst in quiescent bowel, with *DDAH2*. Similarly, in the colon, there was a switch from the correlation with *DDAH1* in inflamed tissue to *DDAH2* in quiescent one. *DDAH1* is the primary enzyme in ADMA catabolism and there is some uncertainty concerning metabolic activity and the role of *DDAH2* [46]. As DDAH enzymes are known mostly as ADMA-metabolizing enzymes and ADMA is primarily implicated in the pathogenesis of cardiovascular diseases [47], little, if not nothing, is known on DDAHs in IBD. Therefore, it would be of interest to address the relevance of presented observations on alterations in PRMT–DDAH interplay in a follow-up study. Increased ADMA and SDMA concentrations in the circulation of IBD patients have been reported in an isolated and unchallenged study of Owczarek et al. [21], in which both metabolites positively correlated with clinical activity of CD and systemic arginine show no significant differences between IBD patients and controls. In the currently examined cohort of patients, ADMA was unaltered while arginine and SDMA were decreased in active CD as compared to both controls and active UC. With respect to SDMA, proinflammatory and pro-oxidative in its nature [47], those results are counterintuitive. Nevertheless, they were supported by weak, yet negative, correlation with CDAI. Unlike ADMA, SDMA is not a substrate for DDAHs, the up-regulated expression of which would have explained the reduction in its level. Indeed, upregulated *DDAHs* correlated with both products of ADMA degradation, that is, citrulline and DMA, which were consistently increased at the systemic level in patients with active and inactive disease. Instead, SDMA is mostly excreted with urine, although its degradation by alanine-glyoxylate aminotransferase 2 (AGXT2) has been shown [47]. An attempt to quantify *AGXT2* expression has been made but the enzyme expression in the bowel was too low. Nonetheless, in addition to *DDAH* overexpression, *DDAH1* was associated with IBD angiogenesis as *VEGFA* and *TP53* were independent predictors of its expression in both quiescent and inflamed bowel. Those findings corroborate recent reports on DDAH1 being a new player in VEGF-dependent angiogenesis in human hepatocellular carcinoma [48] and glioma [49]. DDAH2, in turn, has been implicated in angiogenesis in lung adenocarcinoma [50]. Illogically, however, *HIF1A* displayed a weak but negative association with *DDAH1* in multivariate analysis, an observation that requires further investigation, especially because targeting DDAHs has only recently been proposed as a novel antiangiogenic strategy in cancer [46]. In the present study, in multivariate analysis, *FGF2* was negatively or positively associated with, respectively, *DDAH1* and *DDAH2* expression. FGF2 is implicated in fibrosis, although both profibrotic [51] and antifibrotic [52] activity has been attributed to this growth factor. The *DDAHs* association with *FGF2* warrants further investigation as well, as DDAH2 has recently been shown to alleviate fibrosis associated with diabetic cardiomyopathy [38] and DDAH1 to inhibit the Wnt/*β*-catenin pathway, EMT, and gastric cancer cell migration [53]. Clouding matters, DDAH1 has promoted the migration of prostate cancer cells [54]. As such, *DDAHs* overexpression in IBD might be constructed as beneficial, preventing fibrosis and fibrosis- or cancer-related EMT. Significance of consistently negative association between *DDAH1* expression and local expression of chemokines observed here needs to be addressed in functional studies. Similarly, as observed here, the dependence of *DDAH1* on *TP53* expression and the *DDAH2* inverse relation with cell proliferation markers. Others have shown DDAH1 to promote the proliferation of prostate cancer cells via the ADMA/NO-dependent mechanism [54]. 

Numerous studies have shown NO involvement in IBD pathogenesis (reviewed in [7]) and its overproduction in the inflamed bowel [8,9], whilst NO determination in serum has been proposed as a minimally invasive and rapid test for monitoring of disease activity, particularly in UC [55]. Accelerated NO production in IBD might be attributed to bowel rather than leukocyte overexpression of *NOS2* as no difference between whole blood *NOS2* expression between IBD and healthy controls has been detected [56]. Still, *NOS2* has been upregulated in whole blood of patients with active as compared to inactive disease [56]. Corroborating findings of Colon et al. [25], a stepwise increase in colonic *NOS2* expression from normal to quiescent and to inflamed tissue from UC patients was found. No differences were found in the small bowel, which, however, may result from a lack of truly “normal” reference, available for the colon. The NOS enzymes use arginine for NO synthesis; however, active IBD is reportedly accompanied by downregulated expression of cationic amino acid transporter 2, responsible for arginine up-take, causing mucosal arginine concentrations to be diminished and inversely related to the disease activity [24]. Reduced arginine up-take by inflamed colon may cause amino acid increase in circulation, which would explain a direct correlation between systemic arginine and the severity of endoscopic inflammation observed in this study. Such an explanation is supported by the observation that ADMA and SDMA, competitors for amino acid transporters [10], were independent predictors of systemic arginine, specifically in active UC. Unlike in other studies [21], active CD here was associated with diminished systemic arginine concentrations and, like in other studies [24], no difference in the case of UC was observed. Knockout studies have shown that arginine transporters, and thus arginine, are necessary to reduce mucosal inflammation and the incidence of colitis-associated tumorigenesis [20]. Accordingly, dietary arginine is claimed to be beneficial in animal models of colitis. Correspondingly, its high intake has been linked with restored microbial diversity in the gut, specifically, with an increased prevalence of Bacteroidetes. Consequently, arginine supplementation has led to improved histology and reduced bowel shortening while supplementation with proline or ornithine, amino acids downstream of arginine, had no effect [57].

Kolios et al. [58] reported that epithelial cells are the main source of NOS2 in inflamed colon. However, *NOS2* up-regulation is a hallmark of M1 macrophages and is accompanied by increased expression and secretion of proinflammatory cytokines, that is, IL1*β*, TNF*α*, and IL-12 [59]. Indeed, for *NOS2*, both *IL1B* and *TNFA* expression in the bowel were independent predictors of *NOS2* expression, although while the correlation with *IL1B* was positive, it was negative in the case of *TNFA*. Interestingly, the association was tighter in quiescent and non-inflamed tissue, although tissue healing and remodeling are associated with M2 macrophages, characterized by an upregulated ARG1/2-ODC1 pathway and polyamine synthesis [59]. Therefore, one might expect a positive correlation between *ARGs* and *ODC1* in quiescent tissue. ARG1 is a cytoplasmic isoenzyme and ARG2 a mitochondrial one. While ARG1-generated ornithine may be used in polyamine synthesis, ARG2-synthesized ornithine fuels citrulline or proline formation, with both polyamines and proline facilitating tissue regeneration [11,60]. Still, ornithine can cross the mitochondrial membrane and enrich the cytoplasmic or mitochondrial amino acid pool [11]. This isoenzyme-related distinction in ornithine fate may contribute to a positive association between *ODC1* and *ARG1* observed in some bowel tissues (mostly small bowel) and a lack of association or even a negative correlation with *ARG2* observed in case of quiescent small bowel. In that case, the upregulated *ARG2* may fuel mitochondrial citrulline synthesis on the expanse of cytoplasmic ornithine, downregulating activity of the polyamine synthesis pathway.

Both *ARGs* were upregulated in the colon of IBD patients, and, although without significant differences between inflamed and quiescent tissue, positively and strongly correlated with clinical activity of UC. In the small bowel, *ARG2* was downregulated in inflamed tissue and *ARG1* showed no differences, but its expression level in inflamed tissue positively correlated with CDAI. *ARG1* was particularly strongly correlated with *IL8* expression, a chemoattractant for neutrophils and a proangiogenic factor, and, in quiescent tissue, displayed a negative association with the marker of cell proliferation. *ARG2* in inflamed bowel was independently associated with angiogenic factor *FGF2* in inflamed tissue and *CDKN1A* in non-inflamed. The *ARGs* association with angiogenesis is in line with observations of others, showing their upregulation under hypoxic conditions and in response to IL-8 [60]. Hypoxia-induced *ARG2* overexpression supports cell proliferation in osteosarcoma [61]. Arginase activity has been shown to be necessary for arterial cell proliferation, acting via promoting perivascular M2 macrophage accumulation [62]. 

The ARG enzymes contribute to wound healing by producing ornithine, used either for proline synthesis or in the polyamine generation pathway [60]. It is worth mentioning that putrescine, a direct substrate in polyamine synthesis, can be formed from arginine also via agmatine [63] and therefore can facilitate tissue regeneration by bypassing the ARG and ODC enzymes. Accordingly, agmatinase expression has been demonstrated in the small intestine and immune cells. Moreover, polyamines, being also products of bacterial metabolism, can be directly absorbed from the gut lumen (reviewed in [63]). In the examined cohort of patients, *ODC1* was upregulated in the colon, more markedly in quiescent tissue, but not in the small bowel. ODC1 is a rate-limiting step in the synthesis of polyamines, which play important roles in both physiology and pathology regulating a number of cellular functions; among others, they induce proliferation and migration of intestinal epithelial cells, necessary for repair processes [16,17], but may also induce apoptosis or trigger neoplastic transformation [64,65]. ODC1 inhibition and subsequent polyamine depletion prevented TNFα- induced apoptosis in normal intestinal epithelial cells [16]. Accordingly, *ODC1* expression in studied patients was independently associated with *IL1B* in quiescent bowel and with proapoptotic *BAX* in inflamed bowel. However, the macrophage-derived enzyme has been shown to impair M1 responses and, consequently, exacerbate epithelial injury-associated colitis and promote colitis-associated cancer [66]. *ODC1* overexpression may increase susceptibility to neoplastic transformation as polyamines are involved in epigenetic regulation of gene expression by interacting with histone acetyltransferases, deacetylases, and demethylase and thus affecting chromatin state and global transcription [67]. Targeting ODC1 is currently being tested as a chemoprevention strategy in CRC, which, however, is associated with adenoma-carcinoma transformation [13,14,15] and not inflammation-induced carcinogenesis.

Citrulline is considered a marker of intestinal function in certain conditions and its decrease in circulation has been reported in Crohn’s disease (reviewed in [22]). However, its serum concentrations are believed to depend mostly on amino acid production by the enterocytes [22,23] and others have reported normal citrulline concentration in CD patients except for those with extensive involvement of the small bowel and/or following significant intestinal resection [23]. Interestingly, the source of citrulline in human enterocytes remains uncertain, although studies with stable isotopes seem to point at glutamine as its precursor (reviewed in [23]). Serum concentrations of the amino acid would depend not only on the rate of its synthesis in the gut but also on the efficiency of citrulline transporters, unknown in IBD, and on the amino acid uptake by the kidneys [23]. Here, systemic citrulline was elevated, which, as already mentioned, is consistent with concomitantly elevated DMA and up-regulated expression of DDAHs, observed both in small and large bowel. In turn, no correlation with *NOS2*, *ARG2*, or *ODC1*, controlling alternative routes of citrulline synthesis, could be found. 

While the present study sheds new light on the L-arginine/NO pathway in IBD, it has several limitations that ought to be addressed. Firstly, in view of the poor correlation between local enzyme expression and systemic metabolites, the observed alterations in transcriptional patterns of pathway enzymes need to be confirmed at the protein level and preferably on a larger set of samples. The possible contribution of peripheral leukocytes to the systemic concentrations of analyzed metabolites and the status of pathway enzymes should be investigated as well. Secondly, evaluation of other metabolites such as ornithine, agmatine, and polyamines and other relevant enzymes such as argininosuccinate synthase, argininosuccinate lyase, or ornithine transcarbamylase and amino acid transporters would allow for a fuller understanding of metabolite flux. Thirdly, the metabolic part of the study revealed pathway alterations in irritable bowel syndrome, distinct from those observed in IBD, as well as differences between CD and UC. Those observations are interesting from a clinical point of view as they may imply that L-arginine/NO pathway metabolites have the potential to be employed in differential diagnosis as biomarkers. Owing to the similarity of symptoms, differentiating CD from UC as well as IBD from IBS is difficult and requires extensive diagnostics based on invasive imaging tests. Therefore, non-invasive blood-based biomarkers would be welcome to limit the number of unnecessary colonoscopies. However, although very promising, the results presented in this study had only preliminary character due to a low number of observations. Therefore, they require confirmation on a larger population. As such, the issue of their possible diagnostic utility has not been addressed in the current study.

## 4. Materials and Methods 

### 4.1. Patients

#### 4.1.1. Metabolomic Analysis

Study population for metabolic analysis consisted of 158 individuals: 52 CD patients (40 with active disease and 12 with inactive), 48 UC patients (34 with active disease and 15 with inactive), 18 patients with IBS, and 40 controls. Patients with IBD or IBS were diagnosed and treated in the Department of Gastroenterology and Hepatology or in the First Dept. and Clinic of General, Gastroenterological and Endocrinological Surgery of Wroclaw Medical University. Individuals with indeterminate colitis or the co-existence of other severe systemic diseases, malignancies, liver diseases, or pregnancies were not enrolled. The clinical activity of the diseases was assessed using Crohn’s Disease Activity Index (CDAI), with CDAI ≥150 indicative of active CD, and Rachmilewitz index (RI), with RI ≥6 indicative of active disease. Severity of bowel inflammation in UC patients was assessed using Mayo endoscopic score. With few exceptions, IBD patients were treated with derivatives of 5’-aminosalicylate (5’-ASA). Control sera from apparently healthy blood donors were kindly provided by the Regional Center of Blood Donation and Therapy, Wroclaw, Poland. Demographic characteristics of the study population are given in Table 6.

#### 4.1.2. Transcriptional Analysis

IBD patients (*n* = 44), admitted to the First Dept. and Clinic of General, Gastroenterological and Endocrinological Surgery of Wroclaw Medical University due to the ineffectiveness of pharmacological therapy or the disease complications such as perforation, obstruction, fistula, abscess, or in order to complete further stages of previous surgery procedure, were enrolled. Together, 91 samples were obtained: 51 from small bowel (16 of inflamed tissue from CD patients, 18 of quiescent tissue from CD patients, and 17 of normal tissue from UC patients) and 40 from large bowel (nine of inflamed tissue from CD patients, 15 of inflamed tissue from UC patients, and 16 of quiescent tissue from CD patients). All enrolled UC patients had pancolitis and there was no matched quiescent large bowel tissue to be obtained. Additionally, normal large bowel tissue samples from patients undergoing polypectomy were collected. Only samples from patients whose polyps were subsequently verified in histopathological examination as hyperplastic polyps or tubular adenomas, thus with the lowest potential for malignancy, were used in current study as reference (*n* = 20; *n* = 11 for *PRMT2* determination). Demographic characteristics of the study population are given in Table 7.

For a subset of 28 patients, paired blood samples for metabolomic analysis and tissue samples for transcriptomic analysis (60 samples, as from each patient one to three samples from different sub-locations and/or of different statuses were collected) were available.

### 4.2. Ethical Considerations

The study protocol was approved by the Medical Ethics Committees of Wroclaw Medical University (KB-575/2011 from 10 November 2011 and #KB-247/2018 from 24 April 2018) and the study was conducted in accordance with the Helsinki Declaration of 1975, as revised in 1983, and informed consent has been obtained from all patients.

### 4.3. Analytical Methods

#### 4.3.1. Sample Collection and Preparation

Blood (7.5 mL) was drawn after overnight fasting into serum-separator tubes, clotted for 30 min, and centrifuged (15 min, 10 °C, 720 × *g*). Serum was collected, aliquoted, and stored frozen at −80 °C until examination.

Bowel samples were collected intraoperatively, rinsed with PBS, soaked in RNAlater solution (Ambion Inc., Austin, TX, USA), and stored at −80 °C until ribonucleic acid (RNA) isolation.

#### 4.3.2. Liquid Chromatography Tandem Mass Spectrometry (LC-MS/MS)

Water, acetonitrile, and methanol (LC-MS grade) were obtained from Merck Millipore (Warsaw, Poland). L-arginine, L-citrulline, ADMA, SDMA, hydrochloride salts of unlabeled DMA, hexadeutero-dimethylamine (D6-DMA, 99%), benzoyl chloride (BCl), sodium tetraborate, and formic acid (FA; HPLC grade) were purchased from Sigma Aldrich (Poznan, Poland). Isotope labeled ADMA (2,3,3,4,4,5,5-D7-ADMA, 98%), and L-arginine:hydrochloric acid (D7-arginine, 98%) were purchased from Cambridge Isotope Laboratories (Tewksbury, MA, USA). Leucine–enkephalin was obtained from Waters (Warsaw, Poland).

Serum concentrations of L-arginine/NO pathway metabolites were measured on a Xevo G2 quadrupole time-of-flight (Q-TOF) instrument (Waters, Milford, MA, USA) by stable isotope dilution liquid chromatography tandem mass spectrometry (LC–MS/MS), using a method developed in the laboratory of Medical Biochemistry Department [68]. In short, 100 μL of sera or calibration sample, 10 µL of internal standard solution in water (20 µM D7-ADMA, 50 µM D6-DMA, and 100 µM D7-arginine), and 50 µL of borate buffer (0.025 M Na_2_B_4_O_7_·10H_2_O, 1.77 mM sodium hydroxide (NaOH), pH = 9.2) were vortexed (1 min, 1100 rpm, 25 °C) in polypropylene microtubes. Subsequently, 400 µL of acetonitrile and 10 µL of 10% BCl in acetonitrile were added and vortexed again (5 min, 1100 rpm, 25 °C). Following derivatization, samples were centrifuged (7 min, 4 °C, 15,000 × *g*) and 100 µL aliquots of supernatants were transferred into chromatographic glass vial with 300 µL of water for subsequent LC–MS analysis, carried out using a nanoAcquity ultra performance liquid chromatography (UPLC) system, equipped with an Acquity HSS T3 column (50 mm × 1.0 mm, 1.75 μm) with 0.22 μm membrane inline filter (Waters). Total run time of the method was 10 min with flow rate of 80 µL/min. Mobile phase A consisted of 0.1% FA in water, while mobile phase B consisted of 0.1% FA in methanol. For ADMA and SDMA isomers separation, the following gradient was applied: 11% B for 0–1 min, 11%–13% B for 1–2 min, 13%–60% B for 2–5 min, 60%–90% B for 5–5.5 min, 90% B for 5.5–6 min and 90%–11% B for 6–6.05 min. The sample injection volume was 2 μL. Mass spectra for the compounds were acquired in a Xevo G2 Q-TOF mass spectrometer (Waters) in positive ion mode electrospray ionization (ESI). The MS operating conditions were as follows: capillary voltage, 3000 V; cone voltage, 40 V; source temperature, 120 °C; cone gas flow, 85 L/hour; desolvation temperature, 350 °C; desolvation gas flow, 800 L/hour. Data acquisition was carried on MassLynx software (Waters) using the following ions (*m*/*z*): 279.1457, 286.1749, 307.1717, 314.2076, 280.1297, 150.0919, and 156.1113 for L-arginine, D7-arginine, ADMA, SDMA, D7-ADMA, L-citrulline, DMA, and D6-DMA, respectively. Standard calibration curves were prepared using the following concentration ranges: 5–250 µM for arginine, 0.05–2.5 µM for ADMA and SDMA, 1–50 µM for citrulline, and 0.14–7.0 µM for DMA. The method is characterized by the following intra- and inter-assay CVs: 1.6% and 3.3% (L-arginine), 3.2% and 3.1% (L-citrulline), 1.8% and 5.9% (DMA), 7.5% and 9.4% (ADMA), and 6.4% and 7.1% (SDMA) and was successfully used in patients with hematological malignancies [69], neurodegenerative disorders [70], and cardiometabolic diseases [71].

#### 4.3.3. Reverse-Transcription Quantitative (Real-Time) Polymerase Chain Reaction (RT-qPCR)

Tissue samples (~40 mg) were homogenized using Fastprep 24 Homogenizer (MP Biomedical, OH, USA) in PureLink™ RNA Mini Kit lysis buffer (Thermo Fisher Scientific, Waltham, MA, USA) with added β-mercaptoethanol (Sigma-Aldrich, St.Louis, MO, USA). RNA was isolated using phenol-chloroform extraction and purified using PureLink™ RNA Mini Kit. Genomic deoxyribonucleic acid (DNA) was removed by DNase treatment (PureLink™ DNase Set). RNA was quantified using NanoDrop 2000 (Thermo-Fisher Scientific). Its purity was determined by absorbance ratios at 260, 280, and 230 nm while integrity was tested using LabChip microfluidic technology (Experion platform) and Experion RNA StdSens analysis kits (BioRad, Herkules, CA, USA) and expressed as RNA quality indicator (RQI) score (RQI = 1 indicative of degraded and RQI = 10 of intact RNA). Only RNA with RQI ≥7 was used in subsequent steps.

The amount of 500 ng of RNA per reaction mixture (20 µL) was reversely transcribed using C1000 termocycler (BioRad) and iScript™ cDNA Synthesis Kit (BioRad) according to the manufacturer’ instructions. Matching negative transcription controls (“no-RT”) were prepared alongside.

Quantitative (real-time) PCR (qPCR) were conducted using a CFX96 Real-Time PCR instrument (BioRad) and SsoFast EvaGreen^®^ Supermix (BioRad). The following cycling conditions were applied: 30 s activation at 95 °C, 5 s denaturation at 95 °C, annealing/extension for 5 s at 61 °C, 40 cycles, followed by melting step (60–95 °C with fluorescent reading every 0.5 °C). Reaction mixture (20 µL) contained 2 µL of cDNA at 1:5 dilution, 10 µL of 2 × SsoFast EvaGreen^®^ Supermix, 1 µL of each 10 nM forward and reverse target-specific primers (Genomed, Poland), and water. The primers’ specificities were tested by melting curve analysis and an electrophoresis in a high-resolution agarose (SeaKem LE agarose from Lonza, Switzerland) in tris/borate/ethylenediaminetetraacetic acid (TBE) with SYBR Green (Lonza) detection. Primers’ sequences are listed in Table 8.

Technical replicates were averaged prior analysis. Geometric mean of all Cq values in a given analysis was obtained and subtracted from sample Cq (ΔCq) then linearized by 2^^ΔCq^ conversion and normalized to reference: a geometric mean of *PPIA* and *RPLP0*. The obtained values are referred to as a normalized relative quantity (NRQ) [72] and subjected to statistical analysis. Reference gene selection was based on previous results, showing *PPIA* and *RPLP0* the most suitable pair of genes for studies on bowel tissues [73].

In addition, data on tissue expression of other genes, namely, *BAX*, *CCL2*, *CCL3*, *CCL4*, *FGF2*, *HIF1A*, *IL8*, *IL1B*, *CDKN1A*, *TP53*, *PCNA*, *TNFA*, and *VEGFA* were retrieved from previous studies for the purpose of correlation analysis [74,75].

### 4.4. Statistical Analysis

Data distribution was tested using the Kolmogorov–Smirnov test and homogeneity of variances using the Levene test; log-transformation was used if appropriate (with non-normally distributed data and/or non-homogenous variances) and indicated while presenting individual results. Depending on the distribution and homogeneity of variances, data are presented as means, geometric means, or medians with 95% confidence interval and analyzed using one-way ANOVA with the Student–Newman–Keuls post hoc test or the Kruskal–Wallis *H* test with Conover’s post hoc test. Frequency analysis was conducted using Fisher’s exact test (2 × 2 tables) and *χ*^2^ test (2 × 3 tables). Correlation analysis, depending on data character and distribution, was conducted using Spearman’s rank test or Pearson’s correlation test and results were presented as correlation coefficients *ρ* or *r*, respectively. Multiple least squares regression analysis (stepwise method) was used to identify independent predictors of gene expression with *p* < 0.05 as a criterion for entering a variable into the regression model and *p* > 0.1 as a criterion for removing a variable. Multiple regression results are presented as partial correlation coefficients (r_p_) for independent predictors and coefficient of determination (R^2^) together with corresponding *F* statistics and probability. Power analysis was conducted to determine the smallest sample size that is suitable to detect the effect of a given test at the desired level of significance (type I error 0.05 and type II error 0.2). All tests were two-sided and *p* < 0.05 was considered statistically significant. All analyses were conducted using MedCalc Statistical Software version 19.1.5 (MedCalc Software bv, Ostend, Belgium; https://www.medcalc.org; 2020).

## 5. Conclusions

IBD is associated with deregulated expression of enzymes associated with the L-arginine/NO pathway as well as with altered patterns of their interplay, present in both inflamed and quiescent bowel tissue, which reflect the severity of local inflammation and angiogenic potential. Except for a positive correlation between *DDAHs* and their reaction products citrulline and DMA, there is rather a poor translation between the bowel expression of pathway enzymes and systemic concentrations of their metabolites. 

Further studies are needed to explain the role of upregulated expression of *PRMTs* and *DDAHs* in IBD and their potential contribution to IBD-related angiogenesis, fibrosis, and EMT, as well as their suitability as novel targets for pharmacotherapy in IBD and to address the suitability of metabolite determination for differential diagnosis in IBD.

## Figures and Tables

**Table 1:** L-arginine/NO pathway metabolites in IBD and IBS.

Metabolite	Controls (*n* = 40)	IBS (*n* = 18)	IBD (*n* = 100)	*p*-value
L-arginine [µM]	157.1 (144–177)	142.6 (122–173)	147.7 (137–160)	0.084
L-citrulline [µM]	42.7 (37.9–49.8) ^a,b^	113.7 (65.1–153.7) ^b,c^	74.2 (58.1–85.4) ^a,c^	<0.00001
ADMA [µM]	0.603 (0.57–0.64)	0.532 (0.50–0.63)	0.583 (0.55–0.61)	0.280
SDMA [µM]	0.494 (0.47–0.54) ^a^	0.429 (0.39–0.45) ^b,c^	0.468 (0.45–0.49) ^a^	0.016
DMA [µM]	50.37 (45–53.6) ^a,b^	71.06 (62.5–87) ^c^	69.14 (63.1–74)^c^	<0.00001

Data were not normally distributed and are presented as medians (95% confidence interval) and analyzed using the Kruskal–Wallis *H* test. IBD, inflammatory bowel disease; IBS, irritable bowel syndrome; *n*, number of observations; ADMA, asymmetric dimethylarginine; SDMA, symmetric dimethylarginine; DMA, dimethylamine; ^a^ significantly different from IBS; ^b^ significantly different from IBD; ^c^ significantly different from controls.

**Table 2:** Systemic concentrations of L-arginine/NO pathway metabolites in reference to IBD phenotype and clinical activity.

Metabolite	Controls (*n* = 40)	IBS (*n* = 18)	IBD (*n* = 100)				*p*-value
			Active CD (*n* = 40)	Inactive CD (*n* = 12)	Active UC (*n* = 33)	Inactive UC (*n* = 15)	
L-arginine [µM]	157.1 (144–177) ^a^	142.6 (122–173)	128.5 (107–148) ^b,c^	167.9 (113–179)	163.9 (143–184) ^a^	142.5 (129–160)	0.006
L-citrulline [µM]	42.7 (37.9–49.8) ^a,c–f^	113.7 (65.1–153.7) ^a–c^	68.7 (54.8–90.3) ^b,e^	90.9 (23.5–155) ^b^	65.9 (34.2–86.4) ^b,e,f^	80.5 (56.7–148) ^b,c^	<0.0001
ADMA [µM]	0.603 (0.57–0.64)	0.532 (0.50–0.63)	0.565 (0.53–0.60)	0.600 (0.54–0.69)	0.591 (0.55–0.63)	0.584 (0.46–0.68)	0.555
SDMA [µM]	0.494 (0.47–0.54) ^a,e^	0.429 (0.39–0.45) ^b–d^	0.437 (0.41–0.47) ^b,c^	0.485 (0.44–0.59) ^e^	0.520 (0.47–0.60) ^a,e^	0.457 (0.42–0.50)	0.003
DMA [µM]	50.37 (45–53.6) ^a,c–f^	71.06 (62.5–87) ^b,d^	70.9 (59.7–77.9) ^b,d^	60.5 (49.4–72.1) ^a–c,e^	73.9 (65.2–77.5) ^b,d^	64.8 (47.1–90.3) ^b^	<0.00001

Data were not normally distributed and are presented as medians with 95% confidence interval and analyzed using the Kruskal–Wallis *H* test. IBD, inflammatory bowel disease; CD, Crohn’s disease; UC, ulcerative colitis; IBS, irritable bowel syndrome; *n*, number of observations; ADMA, asymmetric dimethylarginine; SDMA, symmetric dimethylarginine; DMA, dimethylamine; ^a^ significantly different from active CD; ^b^ significantly different from controls; ^c^ significantly different from active UC; ^d^ significantly different from inactive CD; ^e^ significantly different from IBS; ^f^ significantly different from inactive UC.

**Table 3:** Arginine association with Mayo endoscopic score.

Mayo Endoscopic Score (N)	Arginine	*p*-value
0 (10)	139.1 (118–161) ^a^	0.040
1 (8)	159.7 (132–188)	
2 (9)	163.5 (140–187)	
3 (7)	190.8 (150–232) ^b^	

Data were normally distributed and are presented as means with 95% confidence interval (CI) and analyzed using one-way analysis of variance (ANOVA). N, number of observations; ^a^, significantly different from score 3; ^b^, significantly different from score 0.

**Table 4:** L-arginine/NO pathway-associated enzymes in inflamed and quiescent small bowel.

Gene	CD_i (*n* = 16)	CD_q (*n* = 18)	UC_*n* (*n* = 17)	*p*-value
*ARG1* [NRQ]	0.60 (0.25–1.42)	0.81 (0.36–1.82)	0.83 (0.38–1.83)	0.804
*ARG2* [NRQ]	0.67 (0.42–1.09) ^a,b^	2.83 (1.84–4.34) ^c^	1.60 (0.83–3.09) ^c^	0.001
*DDAH1* [NRQ]	0.92 (0.52–1.64)	1.90 (1.21–2.99) ^b^	0.65 (0.30–1.42) ^a^	0.032
*DDAH2* [NRQ]	1.05 (0.89–1.24) ^b^	1.08 (0.85–1.37) ^b^	0.70 (0.61–0.81) ^a,c^	0.002
*NOS2* [NRQ]	1.05 (0.42–2.66)	1.32 (0.47–3.79)	1.50 (0.50–4.48)	0.866
*ODC1* [NRQ]	1.0 (0.82–1.22)	1.03 (0.88–1.21)	0.89 (0.72–1.1)	0.484
*PRMT1* [NRQ]	1.0 (0.84–1.2)	1.21 (0.92–1.61) ^b^	0.81 (0.66–0.99) ^a^	0.036
*PRMT2* [NRQ]	1.16 (0.98–1.39) ^b^	1.22 (0.89–1.68) ^b^	0.71 (0.55–0.92) ^a,c^	0.005
*PRMT5* [NRQ]	1.01 (0.87–1.16)	1.1 (0.94–1.3) ^b^	0.81 (0.66–0.98) ^a^	0.024

Data were normally distributed following log-transformation and are presented as geometric means of normalized relative quantities (NRQ) with 95% confidence interval and analyzed using one-way ANOVA. ARG, arginase; DDAH, dimethylarginine dimethylaminohydrolase, NOS, nitric oxide synthase; ODC, ornithine decarboxylase; PRMT, arginine N-methyltransferase; CD_i, inflamed tissue obtained from patients with Crohn’s disease; CD_q, quiescent tissue obtained from patients with Crohn’s disease; UC_n, normal small bowel tissue obtained from patients with ulcerative colitis; ^a^ significantly different from CD_q; ^b^ significantly different from UC_n; ^c^ significantly different from CD_i.

**Table 5:** L-arginine/NO pathway-associated enzymes in inflamed and quiescent large bowel.

Gene	CD_i (*n* = 9)	CD_q (*n* = 16)	UC_i (*n* = 15)	Normal (*n* = 20)	*p*-value
*ARG1* [NRQ]	0.79 (0.5–28.7) ^a^	1.18 (0.5–7.2) ^a^	8.86 (1.3–15.4) ^a^	0 (0–0) ^b–d^	<0.0001
*ARG2* [NRQ]	0.96 (0.6–6.4) ^a^	1.4 (0.8–3.0) ^a^	1.45 (0.7–2.4) ^a^	0 (0–0) ^b–d^	<0.0001
*DDAH1* [NRQ]	0.48 (0.1–2.9) ^c^	2.75 (0.9–4.7) ^a,b,d^	0.72 (0.2–2.5) ^c^	0.3 (0.2–0.6) ^c^	0.002
*DDAH2* [NRQ]	1.57 (1.2–3.1) ^a,d^	1.54 (1.2–1.9) ^a,d^	0.93 (0.8–1.3) ^a–c^	0.31 (0.1–0.4) ^b–d^	<0.0001
*NOS2* [NRQ]	0.21 (0.05–1.2) ^a,d^	0.74 (0.2–1.5) ^a,d^	7.96 (1–24.2) ^a–c^	0.07 (0.04–0.1) ^b–d^	<0.0001
*ODC1* [NRQ]	1.27 (0.9–2.1) ^a,c^	2 (1.6–2.3) ^a,b^	1.72 (1.4–2.3) ^a^	0.02 (0.01–0.03) ^b–d^	<0.0001
*PRMT1* [NRQ]	1.68 (0.8–3.7) ^a^	1.76 (1.3–2) ^a^	1.33 (1.2–1.8) ^a^	0.08 (0.05–0.2) ^b–d^	<0.0001
*PRMT2* [NRQ]	1.55 (1–5.4) ^a^	1.7 (1.4–3.1) ^a,d^	1.42 (0.9–1.7) ^a,c^	0 (0–0) ^b–d^	<0.0001
*PRMT5* [NRQ]	1.09 (0.9–3) ^a^	1.7 (1.5–2) ^a^	1.54 (1.4–1.8) ^a^	0.07 (0–0.2) ^b–d^	<0.0001

Data were normally distributed following log-transformation and are presented as geometric means of normalized relative quantities (NRQ) with 95% confidence interval and analyzed using one-way ANOVA. ARG, arginase; DDAH, dimethylarginine dimethylaminohydrolase, NOS, nitric oxide synthase; ODC, ornithine decarboxylase; PRMT, arginine N-methyltransferase; CD_i, inflamed tissue obtained from patients with Crohn’s disease; CD_q, quiescent tissue obtained from patients with Crohn’s disease; UC_i, inflamed tissue obtained from patients with ulcerative colitis; Normal, normal large bowel tissue obtained from patients undergoing polypectomy; ^a^ significantly different from normal; ^b^ significantly different from CD_i; ^c^ significantly different from CD_q; ^d^ significantly different from UC_i.

**Table 6:** Demographic characteristics of study population: metabolomic analysis.

Parameter	Controls	IBS	IBD	*p*-value ^1^	IBD				*p*-value ^2^
					Active CD	Inactive CD	Active UC	Inactive UC	
N	40	18	100		40	12	34	14	
Sex (F/M)	20/20	10/8	41/59	0.394 ^3^	19/21	4/8	11/22	7/8	0.577 ^3^
Age [yrs.], mean ± SD	35.9 ± 11.5	41.1 ± 14.5	39.7 ± 14.4	0.251 ^4^	36.4 ± 13.7	42.9 ± 14.3	42.4 ± 15.3	40.2 ± 13.4	0.215 ^4^
Patients with active disease [%]					77		71		0.504 ^5^
Activity, mean ± SD					CDAI: 259 ± 89		RI: 10.4 ± 5		

IBS, irritable bowel syndrome; IBD, inflammatory bowel disease; CD, Crohn’s disease; UC, ulcerative colitis; F/M, female to male ratio; yrs., years; SD, standard deviation; CDAI, Crohn’s disease activity index; RI, Rachmilewitz index; ^1^ for comparison between controls and IBS and IBD patients; ^2^ for comparison between controls and patients with IBS, active and inactive CD and active and inactive UC; ^3^ Χ^2^-test; ^4^ one-way ANOVA; ^5^ Fisher’s exact test.

**Table 7:** Demographic characteristics of study population: transcriptomic analysis.

**Parameter**	**Small Bowel**			***p*-value**
	**UC_n**	**CD_q**	**CD_i**	
N	17	18	16	
Sex (F/M)	9/8	7/11	6/10	0.605 ^1^
Age [yrs.], mean ± SD	36.7 ± 11.9	32.9 ± 6.1	31.9 ± 6	0.232 ^2^
	**Large Bowel**			
	**CD_q**	**CD_i**	**UC_i**	
N	16	9	15	
Sex (F/M)	5/11	3/6	8/7	0.409 ^1^
Age [yrs.], mean ± SD	31.9 ± 6.4	31.3 ± 9.3	37.3 ± 12	0.204 ^2^

N, number of samples; F/M, female to male ratio; yrs., years; SD, standard deviation; ^1^ Χ^2^-test; ^2^ one-way ANOVA; UC_n, macroscopically normal small bowel samples obtained from patients with ulcerative colitis; CD_q, quiescent small or large bowel samples obtained from patients with Crohn’s disease; CD_i, inflamed small or large bowel samples obtained from patients with Crohn’s disease; UC_i, inflamed large bowel samples obtained from patients with ulcerative colitis.

**Table 8:** Primers’ sequences.

Symbol	Encoded Protein	Accession No.	Primer Sequence 5′→3′	Amp. Size [bp]
*PPIA ^1^*	Peptidylprolyl isomerase A	NM_021130.3	F: ggcaaatgctggacccaacaca R: tgctggtcttgccattcctgga	161
*RPLP0 ^1^*	Ribosomal protein, large, P0	NM_001002.3	F: tcacaacaagcataccaagaagc R: gtatccgatgtccacaatgtcaag	263
*ARG1 ^1^*	Arginase-1	NM_001244438.2	F: tcatctgggtggatgctcacac R: gagaatcctggcacatcgggaa	130
*ARG2 ^1^*	Arginase-2	NM_001172.4	F: ctggcttgatgaaaaggctctcc R: tgagcgtggattcactatcaggt	119
*NOS2 ^1^*	Inducible nitric oxide synthase	NM_000625.4	F: gctctacacctccaatgtgacc R: ctgccgagatttgagcctcatg	136
*PRMT1 ^1^*	Arginine *N*-methyltransferase-1	NM_001536.5	F: tgcggtgaagatcgtcaaagcc R: ggactcgtagaagaggcagtag	142
*PRMT2 ^1^*	Arginine *N*-methyltransferase-2	NM_206962.4	F: gcagttggacatgagaaccgtg R: aggctctggaagtggacgctaa	129
*PRMT5 ^1^*	Arginine *N*-methyltransferase-5	NM_006109.5	F: ctagaccgagtaccagaagagg R: cagcatacagctttatccgccg	136
*DDAH1 ^1^*	Dimethylarginine dimethylaminohydrolase-1	NM_012137.4	F: atgcagtctccacagtgccagt R: ttgtcgtagcggtggtcactca	151
*DDAH2 ^1^*	Dimethylarginine dimethylaminohydrolase-2	NM_001303007.2	F: ctttcttcgtcctgggttgcct R: ctccagttctgagcaggacaca	136
*ODC1 ^1^*	Ornithine decarboxylase-1	NM_002539.3	F: ccaaagcagtctgtcgtctcag R: cagagattgcctgcacgaaggt	162

Amp., amplicon; ^1^, primer sequences were as proposed by Origene (www.origene.com), validated in silico by Blast analysis. F, forward primer; R, reverse primer.

## Supplementary Materials

Supplementary materials can be found at https://www.mdpi.com/1422-0067/21/5/1641/s1.

## Abbreviations

IBD	Inflammatory bowel disease
IBS	Irritable bowel syndrome
CD	Crohn’s disease
UC	Ulcerative colitis
CDAI	Crohn’s Disease Activity Index
RI	Rachmilewitz index
ADMA	Asymmetric dimethylarginine
SDMA	Symmetric dimethylarginine
DMA	Dimethylamine
PRMT	Arginine N-methyltransferase
DDAH	Dimethylarginine dimethylaminohydrolase
NOS	Nitric oxide synthase
ODC	Ornithine decarboxylase
ARG	Arginase
