# Supplementary material for: Transcriptional and Metabolomic Analysis of L-Arginine/Nitric Oxide Pathway in Inflammatory Bowel Disease and Its Association with Local Inflammatory and Angiogenic Response: Preliminary Findings
Source: Int J Mol Sci. 2020 Feb 28;21(5):1641. doi: 10.3390/ijms21051641 (PMC7084352; doi:10.3390/ijms21051641)
Supplement: Supplementary file 1 [file ijms-21-01641-s001.pdf]

Supplementary Material

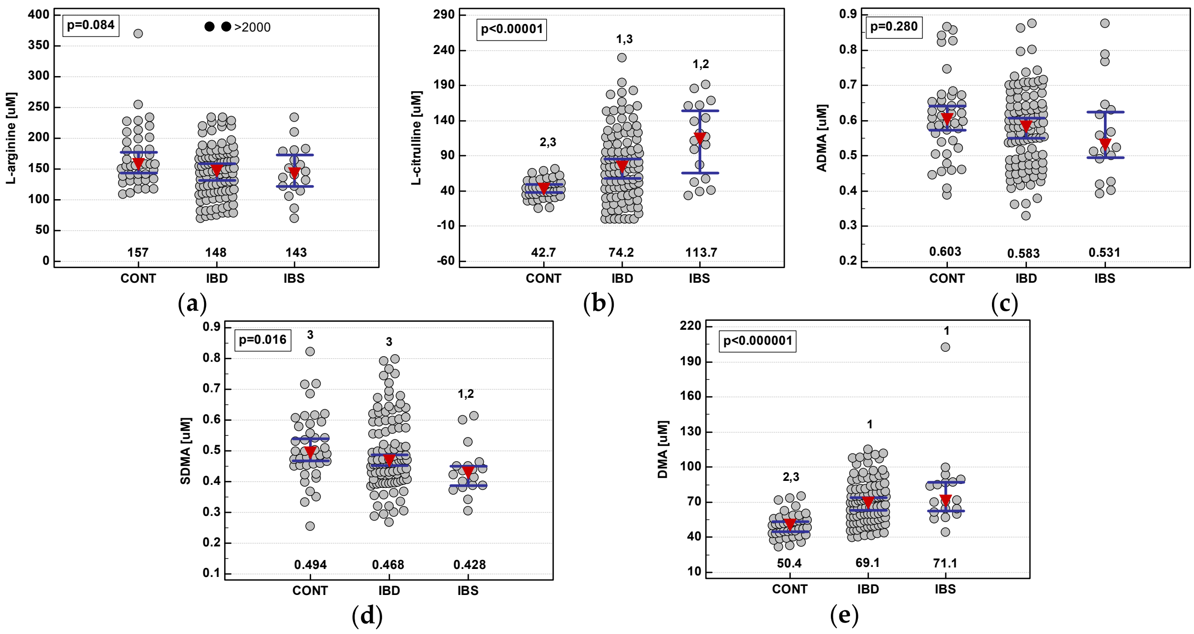

Figure S1. Systemic concentrations of L-arginine/NO pathway metabolites: (**a**) arginine; (**b**) citrulline; (**c**) asymmetric dimethylarginine; (**d**) symmetric dimethylarginine; (**e**) dimethylamine. CONT, healthy controls; IBD, inflammatory bowel disease; IBS, irritable bowel syndrome; 1, significantly different from CONT; 2, significantly different from IBD; 3, significantly different from IBS. Data analyzed using Kruskal-Wallis *H* test and presented as medians (red triangles, numbers below dot-plots) with 95% confidence interval (whiskers).

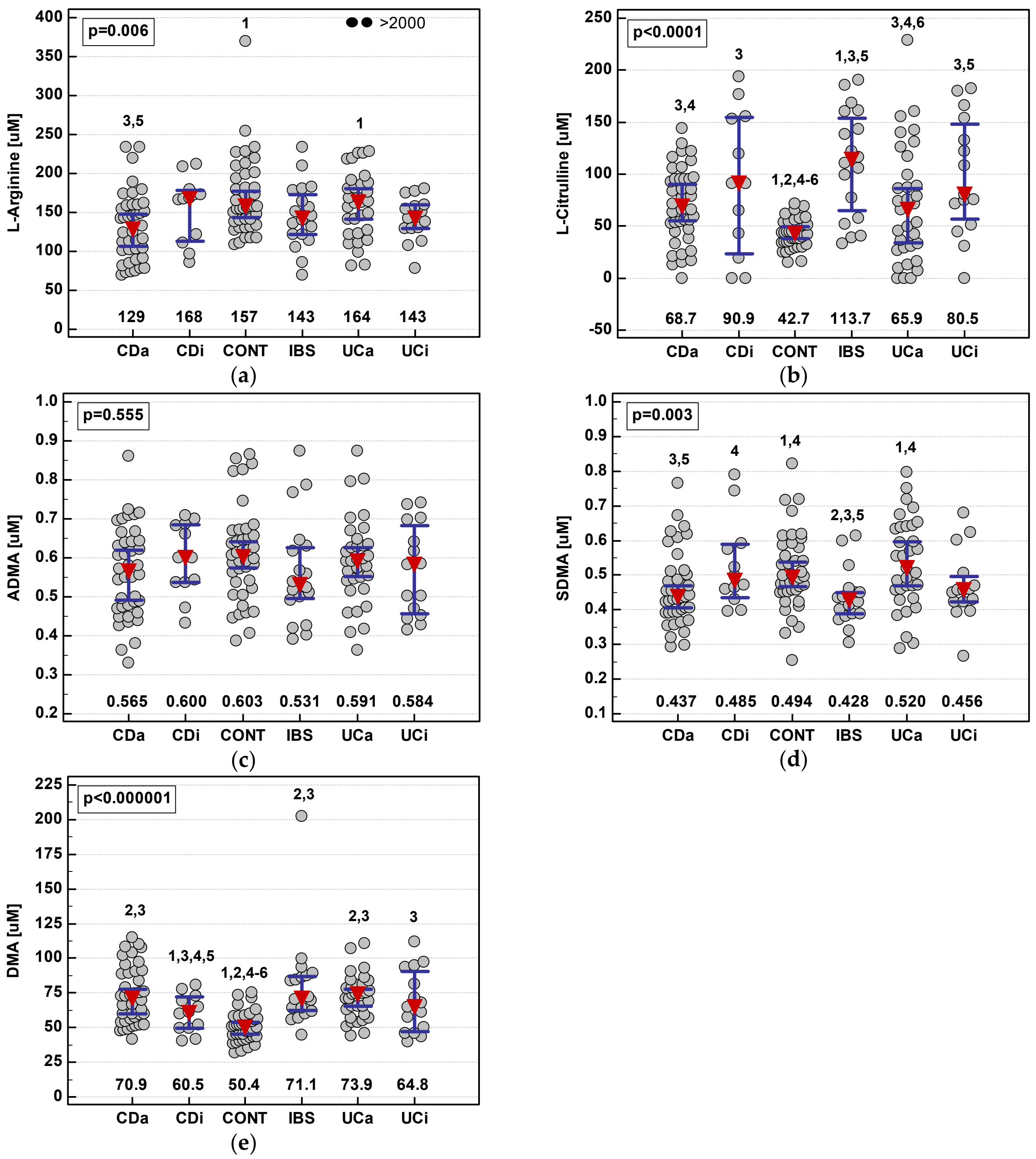

Figure S2. Systemic concentrations of L-arginine/NO pathway metabolites: (**a**) arginine; (**b**) citrulline; (**c**) asymmetric dimethylarginine; (**d**) symmetric dimethylarginine; (**e**) dimethylamine. CDa, active Crohn's disease; CDi, inactive Crohn's disease; CONT, healthy controls; IBS, irritable bowel syndrome; UCa, active ulcerative colitis; UCi, inactive ulcerative colitis; 1, significantly different from CDa; 2, significantly different from CDi; 3, significantly different from CONT; 4, significantly different from IBS; 5, significantly different from UCa; 6, significantly different from UCi. Data analyzed using Kruskal-Wallis *H* test and presented as medians (red triangles, numbers below dot-plots) with 95% confidence interval (whiskers).

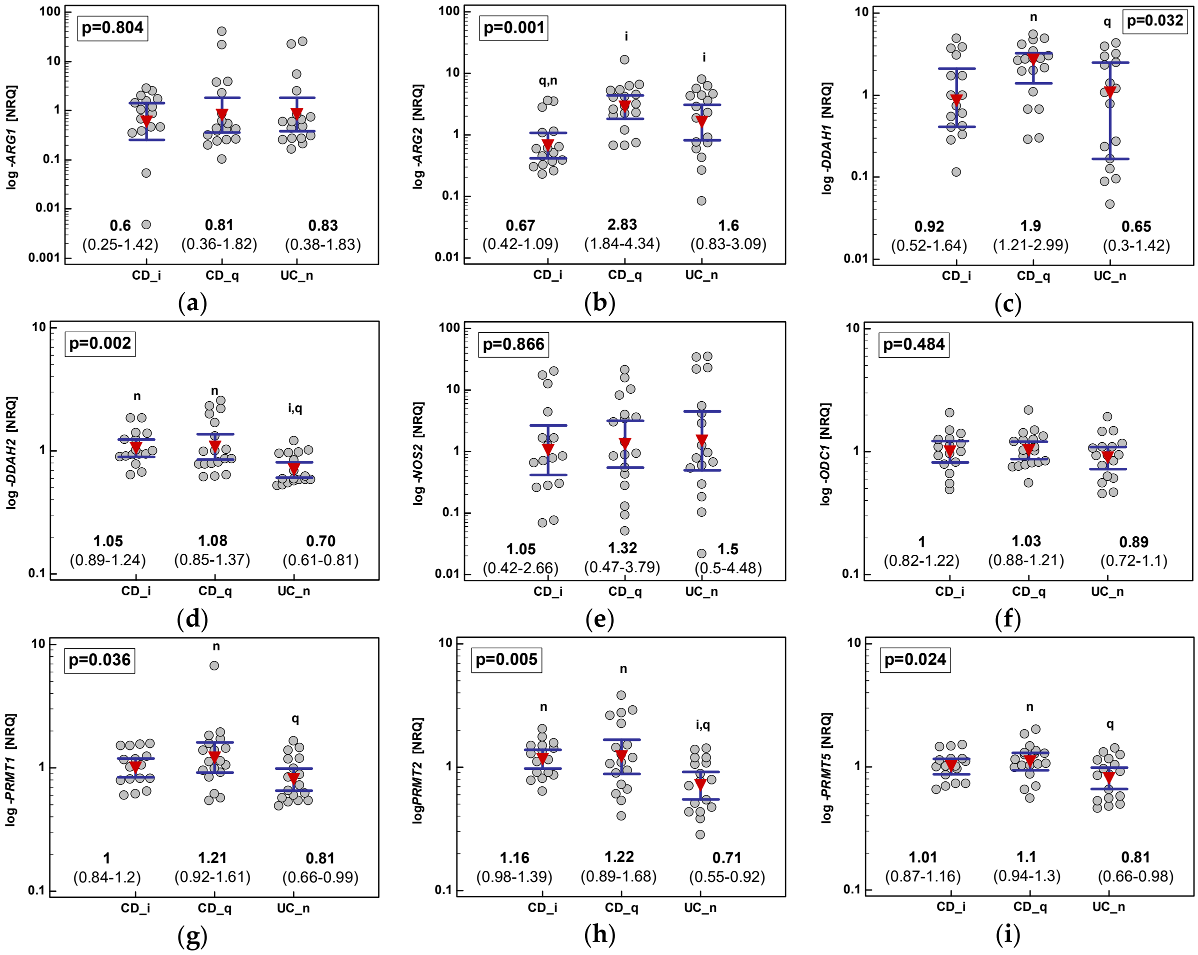

Figure S3. L-arginine/NO pathway-associated enzymes in inflamed and quiescent small bowel: ***(a)*** ARG1; ***(b)*** ARG2; ***(c)*** DDAH1; ***(d)*** DDAH2; ***(e)*** NOS2; ***(f)*** ODC1; ***(g)*** PRMT1; ***(h)*** PRMT2; ***(i)*** PRMT5. Data presented as geometric means of normalized relative quantities (NRQ) with 95% confidence interval (*CI*) and analyzed using one-way ANOVA. CD_i, inflamed tissue obtained from CD patients; CD_q, quiescent tissue obtained from CD patients; UC_n, normal small bowel tissue obtained from UC patients; n, significantly different from UC_n; q, significantly different from CD_q; i, significantly different from CD_i.

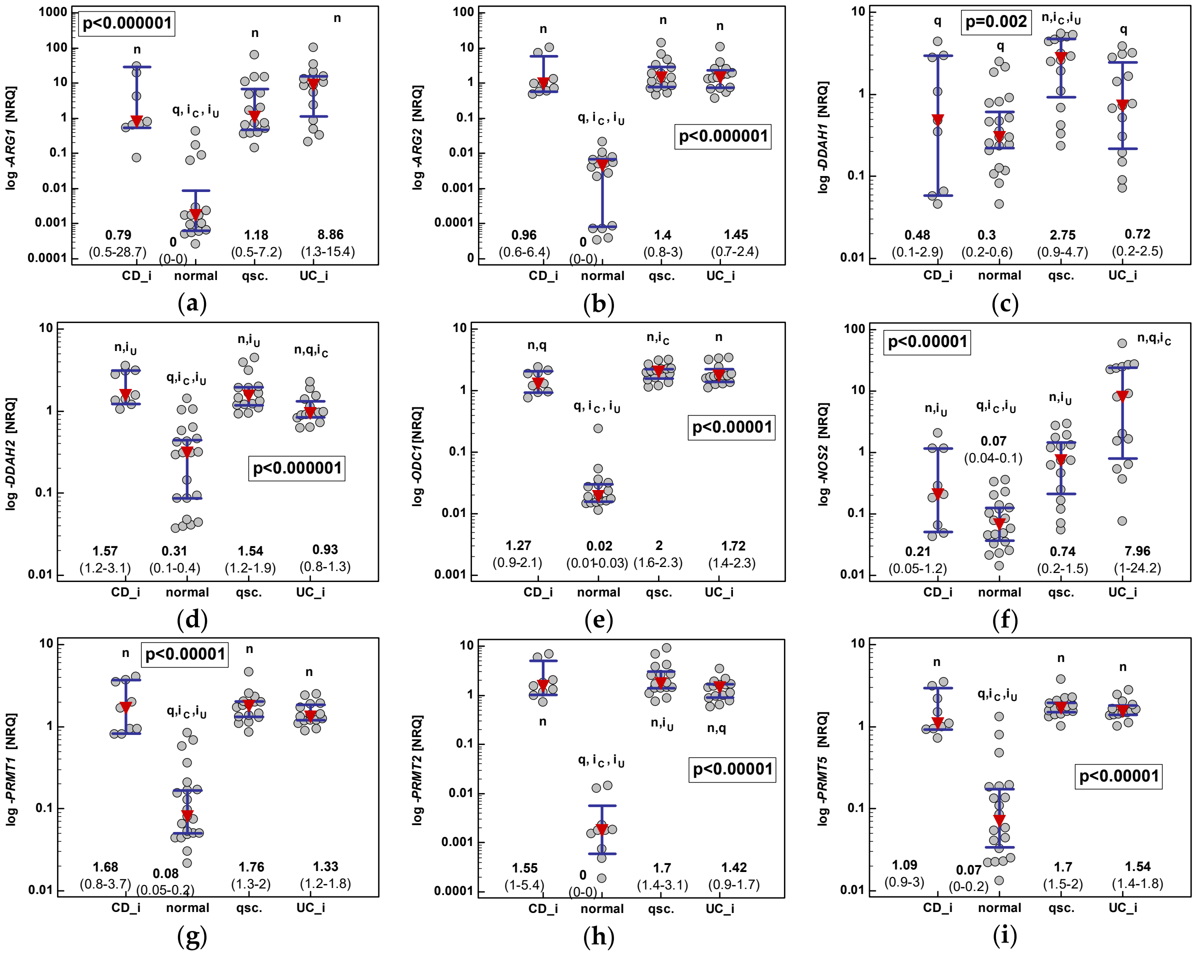

Figure S4. L-arginine/NO pathway-associated enzymes in inflamed and quiescent large bowel: ***(a)*** ARG1; ***(b)*** ARG2; ***(c)*** DDAH1; ***(d)*** DDAH2; ***(e)*** NOS2; ***(f)*** ODC1; ***(g)*** PRMT1; ***(h)*** PRMT2; ***(i)*** PRMT5. Data presented as medians of normalized relative quantities (NRQ) with 95% confidence interval (*CI*) and analyzed using Kruskal-Wallis *H* test. CD_i, inflamed tissue obtained from CD patients; qsc., quiescent tissue obtained from CD patients; UC_i, inflamed tissue obtained from UC patients; normal, normal large bowel tissue obtained from patients undergoing polypectomy; n, significantly different from normal; q, significantly different from qsc.; iC, significantly different from CD_i; iU, significantly different from UC_i.
